# Subcutaneous Allergen Immunotherapy With Hypoallergenic Bet v 1 Compared to Conventional Extract: Poorer Blocking Antibody Capacity Dominated by IgG_1_ Instead of IgG_4_

**DOI:** 10.1111/all.16606

**Published:** 2025-06-02

**Authors:** Lorenz Aglas, Line Kring Tannert, Serge A. Versteeg, Scott A. Smith, Ewa A. Bartko, Mario Wenger, Amin Kraiem, Hannah Widauer, Natália Nunes, Sibile Sinkunaite, Frank Stolz, Laurian Jongejan, Angela Neubauer, Lars H. Blom, Fatima Ferreira, Lars K. Poulsen, Carsten Bindslev‐Jensen, Ronald van Ree

**Affiliations:** ^1^ Institute of Pathophysiology and Allergy Research, Center for Pathophysiology, Infectiology and Immunology Medical University of Vienna Vienna Austria; ^2^ Department of Biosciences and Medical Biology University of Salzburg Austria; ^3^ Odense Research Center for Anaphylaxis Odense University Hospital Odense Denmark; ^4^ Department of Experimental Immunology Amsterdam University Medical Centers Amsterdam the Netherlands; ^5^ Department of Medicine Vanderbilt University Medical Center Nashville Tennessee USA; ^6^ Department of Dermatology and Allergy, Allergy Clinic Copenhagen University Hospital‐Herlev and Gentofte Copenhagen Denmark; ^7^ Vienna Competence Center Biomay AG Vienna Austria; ^8^ Department of Clinical Medicine University of Copenhagen Copenhagen Denmark; ^9^ Department of Dermatology and Allergy Center Odense University Hospital Odense Denmark; ^10^ Department of Otorhinolaryngology Amsterdam University Medical Centers Amsterdam the Netherlands

**Keywords:** AIT, Bet v 1, birch, hypoallergen, IgG_4_

## Abstract

**Background:**

Hypoallergenic recombinant fold‐variants of major allergens have been suggested as safer and more effective AIT candidates. The Bet v 1‐fold variant BM41, with confirmed preclinical hypoallergenicity and increased immunogenicity, was proposed for the treatment of birch pollen allergy.

**Methods:**

We performed a 6‐month randomized, double‐blind, placebo‐controlled first‐in‐human clinical trial with BM41, a licensed birch pollen extract‐based treatment, as the active comparator (AC), and placebo (*n* = 16, *n* = 16, and *n* = 15, respectively). The primary endpoint was safety. Secondary outcomes were Bet v 1‐specific (s)IgE, IgG, IgG_1_, and IgG_4_ responses measured by ImmunoCAP, and sIgE‐blocking activity using mediator release and facilitated antigen binding assays.

**Results:**

Despite SPT‐confirmed hypoallergenicity (~50% compared to natural Bet v 1), more adverse events occurred in response to BM41. Although similar sIgG and sIgG_1_ levels were induced, sIgG_4_ levels increased 3‐fold more in AC compared to the BM41 group. In AC, the sIgG_4_/sIgG_1_ ratio tripled over time, whereas for BM41 it stagnated. BM41 induced efficient serum inhibitory activity for sIgE compared to placebo but was 12%–32% less efficient than AC. Both sIgG_4_ and sIgG_1_ contributed to the blocking effect in AC, while in BM41 both sIgG subclasses showed a lowered functional capacity.

**Conclusion:**

Preclinically established hypoallergenicity of BM41 did not result in a lower number of adverse events. The reduced induction of sIgG_4_ by the fold variant in the course of the treatment was less efficient in blocking sIgE‐mediated responses. This is the first study providing evidence that, instead of a Th1‐favored IgG_1_‐dominated response, “modified Th2”‐skewed IgG_4_‐dominated humoral responses are beneficial in AIT vaccine design.

## Graphical abstract

A 6‐month randomized, double‐blind, placebo‐controlled first‐in‐human clinical trial was conducted in birch pollen allergic patients comparing the Bet v 1‐fold‐variant BM41 with a licensed birch pollen extract‐based treatment, as active comparator, and placebo. Despite SPT‐confirmed hypo‐allergenicity (~50% compared to natural Bet v 1), more adverse events occurred in response to BM41. In BPE‐AC, the sIgG4/sIgG1 ratio tripled over time whereas for BM41 it stagnated. BM41 induced efficient serum inhibitory activity for sIgE compared to placebo but was 12‐32% less efficient than BPE‐AC.

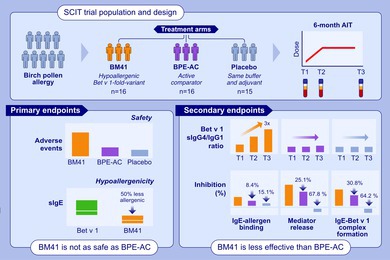

Abbreviations: AIT, allergen immunotherapy; BM41, Bet v 1‐fold‐variant; BPE‐AC, birch pollen extract‐based active comparator; sIg, specific immunoglobulin; SPT, skin prick test.

**Abbreviations**
AIT: allergen‐specific immunotherapy
AUC: area‐under‐the‐curve
BPE: birch pollen extract
BPE‐AC: birch pollen extract‐based active comparator
EBV: Epstein–Barr virus
ELISA: enzyme‐linked immunosorbent assay
FAB: facilitated allergen binding
FAB competition: facilitated allergen binding inhibition assay
FAB inhibition: facilitated allergen binding inhibition assay
FcεRI: high‐affinity IgE receptor I
FcεRII: CD23; IgE affinity receptor II
GMP: good manufacturing practice
Ig: immunoglobulin
IMP: investigational medicine product
iMRA: inhibition MRA
ISAC: immuno solid allergen chip
LOQ: limit of quantification
MedDRA: medical dictionary for regulatory activities
MRA: mediator release assay
OAS: oral allergy symptoms
PCA: principal component analysis
PFAS: pollen food allergy syndrome
RBL‐2H3: rat basophil leukemia cells
s: specific
SAE: severe adverse event
SCIT: subcutaneous allergen immunotherapy
Th: T helper cell
tSPT: titrated skin‐prick‐test

## 1 Introduction

Allergen immunotherapy (AIT) is the only disease‐modifying treatment for IgE‐mediated allergies that can achieve sustained tolerance [1]. Lengthy high‐frequency treatment protocols (3–5 years of monthly injections) and the common occurrence of side effects are the main reasons for > 90% of patients to favor symptom medication over AIT. Not all patients respond to AIT; that is, for birch pollen AIT, only 63% of patients showed an improvement of symptoms, medication scores, and quality of life of higher than 60% compared to before the treatment [2]. There are several approaches to make AIT safer and more effective, including the use of novel adjuvants and routes of administration, as well as the substitution of extract‐based vaccines by recombinant major allergens and hypoallergenic mutants thereof. In this context, recombinant fold variants of major allergens were introduced, acquiring hypoallergenicity due to the modification of conformational IgE epitopes.

A physicochemically induced folding variant of the major birch pollen allergen Bet v 1 was evaluated for safety and efficacy in a Phase II trial, but no increased efficacy was achieved compared to the conventional extract‐based treatment (ClinicalTrials.gov Identifier: NCT00266526). The development was discontinued after an unsuccessful Phase III trial [3]. Other attempts to screen hypoallergenic Bet v 1‐fold variants in AIT trials included recombinant half molecules of the wild‐type allergen and covalently linked trimers of Bet v 1 [4, 5]. Both resulted in the induction of robust allergen‐specific (s)IgG responses, although not dominantly of the IgG_4_ subclass [6], and without significant improvement of combined symptom medication scores compared to placebo [7]. Recombinant wild‐type Bet v 1 proved similarly effective as a licensed extract‐based treatment in reducing rhino‐conjunctivitis symptoms and use of rescue medication [8]. Together, these observations raise several questions: (i) how relevant is an intact structure for the induction of blocking sIgG_4_ and associated treatment effects, and (ii) how important are other extract components potentially having adjuvant activity. Until now, no hypoallergenic recombinant AIT product has obtained an official drug approval by regulatory agencies [9], partly because a clinically relevant improvement could not be demonstrated, partly because companies discontinued development due to the absence of significant improvement compared to conventional extract‐based AIT.

Within the EU‐funded project “BM4SIT – Innovations for Allergy” (www.BM4SIT.eu), we investigated the performance of another Bet v 1‐fold‐variant, BM41, in a subcutaneous, randomized, double‐blind, placebo‐controlled first‐in‐human clinical trial with safety as primary endpoint. BM41 was created by altering five amino acids in the primary sequence of Bet v 1, which resulted in a collapse of the characteristic PR‐10 fold [10]. The fold‐variant was chosen based on three promising preclinically identified criteria: a high degree of (i) in vitro hypoallergenicity [10], (ii) in vivo immunogenicity in rodents and rabbits combined with T helper class 1‐ (Th1‐) skewing effects, and (iii) the functional capacity of the animal sIgG antibodies, induced in vivo, to block Bet v 1‐specific human IgE responses [11, 12]. Preliminary efficacy of BM41 was investigated as secondary endpoint by evaluating established serum biomarkers as surrogate for AIT efficacy [13, 14, 15]. For comparison of safety and efficacy, an established SCIT product with market authorization, Alutard SQ *Betula verrucosa* , was used as active comparator.

## 2 Materials and Methods

### 2.1 Subjects

**FIGURE 1:**
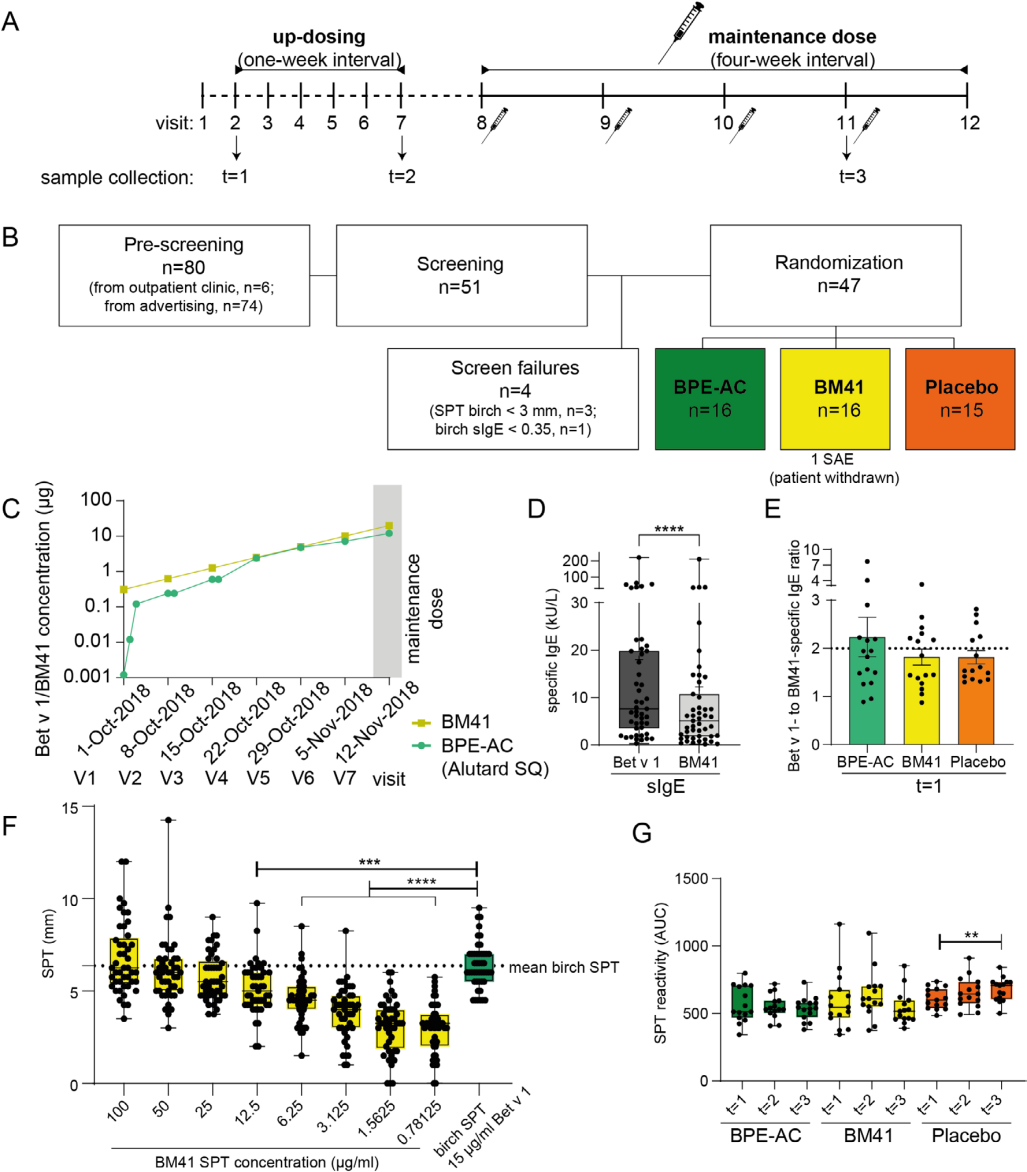
SCIT protocol for administration of BM41 and placebo (A), patient screening and randomization (B), and hypoallergenicity assessment in tSPT and ImmunoCAP. The concentrations of BM41 that were administered were adjusted to the Bet v 1 concentrations contained in BPE‐AC in a cluster up‐dosing regimen (C); V1 = *t* = 1; V7 = *t* = 2. Patients' IgE was significantly binding less to BM41 compared to Bet v 1 in ImmunoCAP (D), and the reactivity of patients' IgE to Bet v 1 was about twice as high as for BM41 in all three treatment groups at *t* = 1 (E). Data from tSPT with BM41 of all patients combined before first SCIT administration (at *t* = 1, *n* = 47) in comparison to results obtained with birch pollen extract SPT solution performed at the inclusion phase of the patients' screening (F). The median of 15 μg/mL Bet v 1 of the birch pollen SPT resulted in a similar wheal size as 100 μg/mL of BM41. The reactivity of the patients to BM41 was investigated using a tSPT at the three various time points of the treatment and the AUC of the dose–response curves were calculated per patient (G). BM41 SPT sizes remained stable in both active treatment groups throughout the trial, while for placebo the area‐under‐the‐curve (AUC) of the tSPT wheal‐and‐flare reactions increased significantly from *t* = 1 to *t* = 3 (pollen season of early flowering trees. BPE‐AC, birch pollen extract‐based active comparator; SAE, severe adverse events; sIgE, specific IgE; tSPT, titrated skin‐prick‐test. **p ≤ 0.01; ***p ≤ 0.001; ****p ≤ 0.0001.

The 6‐month subcutaneous allergen immunotherapy (SCIT) trial with an open comparator was conducted from September 2018 to March 2019 at the Allergy Center, Odense University Hospital, Denmark (Figure 1A, ClinicalTrials.gov Identifier: NCT04912076). Birch pollen allergic patients with moderate to severe symptoms were screened, and 47/51 screened patients were randomized (Figure 1B). Demographic data and clinical response at inclusion can be found in Table S1; inclusion and exclusion criteria can be found in Tables S2 and S3, respectively. Sixteen birch pollen allergic patients were treated with BM41 (maintenance dose 20 μg BM41/0.2 mg/mL aluminum hydroxide, Biomay AG, Vienna, Austria), 16 patients with the birch pollen extract‐based active comparator (BPE‐AC), Alutard SQ ( *Betula verrucosa* , ALK, Horsholm, Denmark), at a maintenance dose 100.000 SQ/mL containing 12 μg Bet v 1 [16], and 15 patients with the adjuvant aluminum hydroxide as control (0.2 mg/mL, Biomay AG), termed placebo in the following. The concentration of BM41 during the up‐dosing phase and in the maintenance dose was adjusted to the Bet v 1 concentration contained in BPE‐AC (Figure 1C). Sera and full blood were collected at visit 2 (before initial SCIT administration), visit 7 (reaching the maintenance dose), and visit 11 (1 month after final SCIT administration), termed *t* = 1, *t* = 2, and *t* = 3 in the following, respectively. The study was approved by the Danish Medicines Agency and the Regional Committees on Health Research Ethics for Southern Denmark (EudraCT number 2018‐001486‐17). Informed consent was signed by all patients.

### 2.2 Safety and Hypoallergenicity Assessment

Detailed information about the reporting of adverse events and the titrated skin‐prick‐test (tSPT) using BM41 can be found in the Supporting Information.

### 2.3 Immunological Assays

Specific IgE, IgG_1_, IgG_4_, and IgG were measured in patients' sera by ImmunoCAP (Thermo Fisher Scientific, Uppsala, Sweden). Detailed information as well as on mediator release assay (MRA), IgE‐facilitated allergen binding (FAB) assay, assays to assess serum inhibitory activity (inhibition enzyme‐linked immunosorbent assay, ELISA; inhibition MRA, iMRA; and FAB inhibition/FAB competition assays), and the IgG_1_‐ and IgG_4_‐depletion protocol can be found in the Supporting Information.

### 2.4 Statistical Analysis

For statistical analysis, either the same time point in different treatment groups (ordinary ANOVA) or different time points within the same treatment group (repeated‐measure ANOVA, or mixed effects analysis in the BM41 group due to the missing data point because of patient withdrawal) were analyzed, and a Tukey's multiple comparisons post hoc test was used. For correlation analysis, a two‐tailed Spearman's rank correlation was used. Due to an unexpected uneven distribution of sIgE among treatment groups, the delta‐value (Δ), that is, the change between time points, was calculated (e.g., Δ = parameter (*t* = 3) − parameter (*t* = 1)) for each patient. Since the Δ‐values were calculated individually for each patient due to the paired design of the clinical trial (three time points per patient), the median of Δ‐values does not necessarily result in the same number as the difference of the medians of a parameter of two time points. The following *p* values were considered significant: *p ≤ 0.05; **p ≤ 0.01; ***p ≤ 0.001; ****p ≤ 0.0001. Details on used statistical tests are provided in the Supporting Information.

## 3 Results

### 3.1 Hypoallergenicity of BM41

Hypoallergenicity of BM41 was confirmed by ImmunoCAP, since the reactivity of patients' IgE to bind to BM41 was significantly lower than to Bet v 1 at beginning of the clinical trial (*t* = 1), with a difference in the medians of 2.54 kU/L (5.12 kU/L vs. 7.66 kU/L, respectively, Figure 1D). Among individual patients, the responsiveness of patients' IgE to BM41 was only half that observed for Bet v 1, as indicated by the Bet v 1‐ to BM41‐sIgE ratio (Figure 1E). For the in vivo hypoallergenicity assessment, the tSPT data using BM41 at *t* = 1 were compared to the birch pollen SPT data at inclusion (Figures 1F,G, S1A–D). Birch pollen SPT, containing 15 μg/mL Bet v 1, resulted in an average wheal size of 6.37 mm (median), whereas the comparable concentration of BM41 (12.5 μg/mL) induced a significantly lower wheal size (5.14 mm).

### 3.2 Safety of BM41

Despite hypoallergenicity, surprisingly, more adverse events (*n* = 45), both immediate and delayed, were reported in the BM41 arm than in placebo (*n* = 11) and the BPE‐AC arms (*n* = 18). Adverse events mainly comprised localized skin reactions at the injection site. Immediate local skin reactions appeared in 8/16 (50%) in the BM41 group, in 2/16 (12.5%) in the BPE‐AC group, and in 1/15 (6.7%) in the placebo group (Table 1). One patient receiving BM41 developed a severe adverse event (SAE) commencing within a few minutes after the administration of the first maintenance dose (*t* = 2) and, thus, was excluded from completing the study. Interestingly, the nature of the symptoms occurring in the BPE‐AC group was similar to those in the placebo group, suggesting that these are mainly local alum‐associated side effects.

**TABLE 1:** Adverse events related to investigational medicine product (IMP).

	AEs reported	BPE‐AC (*n* = 16)		BM41 (*n* = 16)		Placebo (*n* = 16)	
		No. of patients with at least one AE (%)	No. of events	No. of patients with at least one AE (%)	No. of events	No. of patients with at least one AE (%)	No. of events
Severe AE (SAE)	Grade 3 allergic reaction^b^	0 (0%)	0	1 (6.25%)	1	0 (0%)	0
Rhinitis	Rhinitis	1 (6.25%)	1	1 (6.25%)	1	1 (6.7%)	1
Eyes	Itching	1 (6.25%)	1				
	Conjunctivitis			1 (6.25%)	1		
Respiratory tract	Dyspnoea			1 (6.25%)	3		
	Asthma			1 (6.25%)	1		
Skin	Pruritus			1 (6.25%)	3		
	Atopic dermatitis			1 (6.25%)	1		
	Urticaria			1 (6.25%)	1		
	Angioedema (eyes, uvula)			1 (6.25%)	1		
	Immediate local reaction	2 (12.5%)	3	8 (50%)	14	1 (6.7%)	2
	Delayed local reactions^a^	6 (37.5%)	13	6 (37.5%)	19	6 (40%)	8

^a^ 1 delayed local reaction > 8 cm in the BPE‐AC group. Others < 5 cm.

^b^ Patient developed grade 3 allergic reaction including generalized urticaria, angioedema (including of the uvula), asthma, and rhino‐conjunctivitis. As the patient had no adverse events during up‐dosing but the BM41‐sIgE increased by 21% (from 6.24 at *t* = 1 to 7.54 kU/L at *t* = 2) compared to 6% Bet v 1‐sIgE, de‐novo induction of IgE against BM41 might have been the cause of SEA.

**FIGURE 2:**
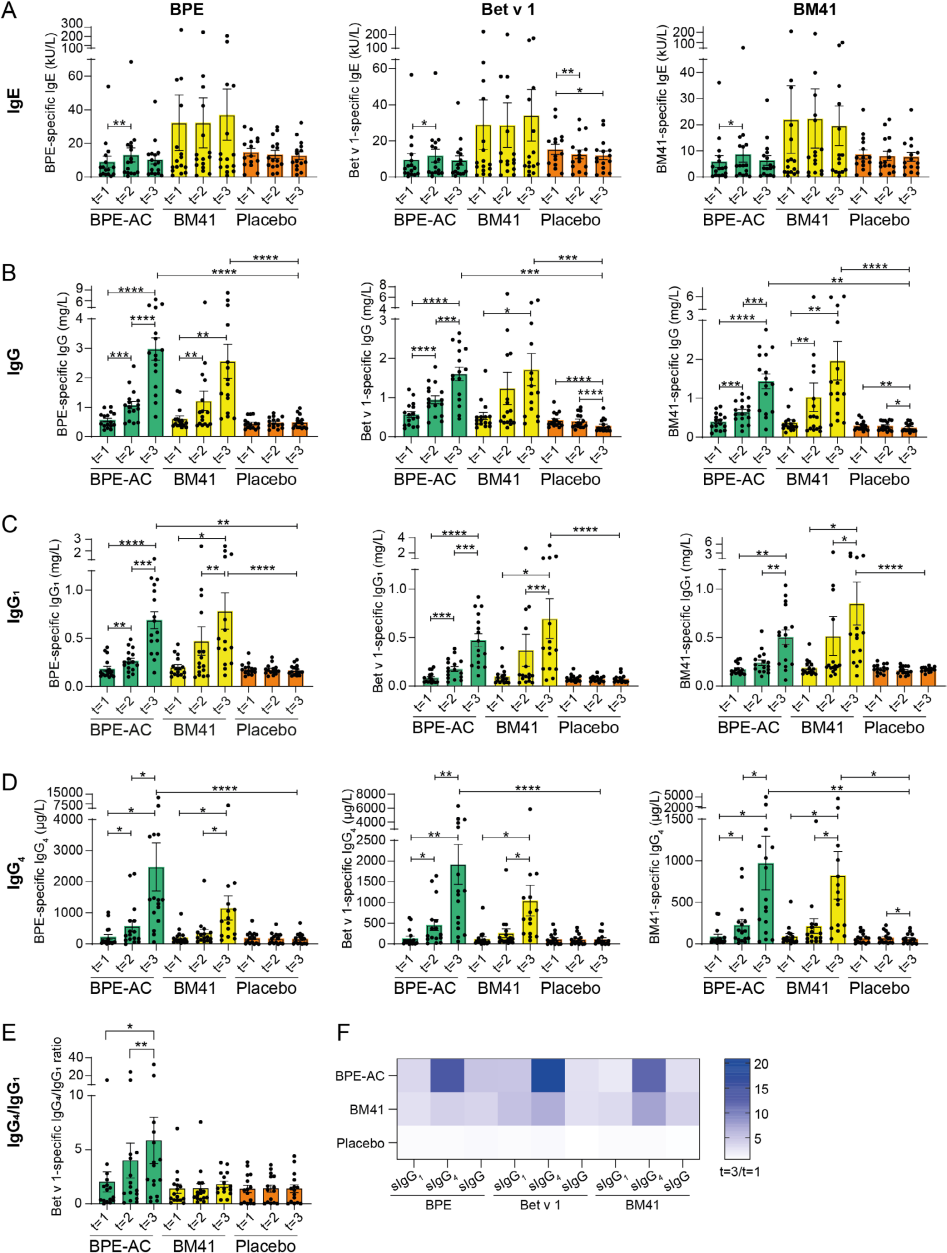
BPE‐, Bet v 1‐ and BM41‐specific (s) IgE (A), IgG (B), IgG_1_ (C) and IgG_4_ levels (D) in sera of SCIT patients treated with either BM41, BPE‐AC or placebo. Placebo treatment showed a small but significant decrease in IgE against Bet v 1 (A). The average fold increase (*t* = 1 to *t* = 3) for BM41 sIgG and sIgG_1_ (B‐C) was higher in the BM41 (4.2‐ and 3.4‐fold, respectively) compared to the BPE‐AC group (3.0‐ and 2.3‐fold, respectively), indicating either that not all BPE‐AC‐induced Bet v 1 sIgG antibodies are cross‐reacting with BM41, and vice versa, or that they are of lower affinity. Data are shown as bar charts (including individual data) with mean and standard error of the mean. Ratios of Bet v 1‐sIgG_4_/IgG_1_ between the different time points (E). The medians of BPE‐, Bet v 1‐ and BM41 sIgG_1_, sIgG_4_ and sIgG fold increase (*t* = 1 to *t* = 3) for all three treatment groups is shown (F). BPE‐AC, birch pollen extract‐based active comparator; s, specific. *p ≤ 0.05; **p ≤ 0.01; ***p ≤ 0.001; ****p ≤ 0.0001.

A possible explanation for the higher number of adverse events in the BM41 group may be that some of the patients randomized to this arm of the study clearly had higher baseline sIgE levels against BPE, Bet v 1, and BM41 (> 40 kU/L), although this did not reach significance on a group level (Figures 2A, S2A–D).

In contrast to BM41, BPE‐AC treatment resulted in a significant but transient increase in sIgE against BPE, Bet v 1, and BM41 during the up‐dosing phase (Figure 2A). Although BM41 did not induce an increase in Bet v 1‐sIgE, the potency of sIgE to trigger mediator release upon Bet v 1 stimulation increased in the course of AIT, which was not observed for BPE‐AC and placebo (Figures S3A,C and S4). However, both active treatments, BM41 and BPE‐AC, induced a downregulation of sIgE‐Bet v 1 complex formation by a median 25.7% and 8.8%, respectively, from *t* = 1 to *t* = 3, most likely due to the induction of blocking antibodies (Figures S3B,D,E and S5). Placebo did not change significantly. The highest values for mediator release and complex formation were observed for the BM41 group, which is in line with the higher baseline sIgE levels observed for this group.

### 3.3 BM41 Induced sIgG More Dominated by sIgG_1_ Than sIgG_4_ Compared to BPE‐AC

BM41 significantly induced sIgG (3.8‐ and 3.0‐fold, median) and sIgG_1_ (2.9‐ and 5.3‐fold) titers against BPE and Bet v 1, respectively, to the same extent as BPE‐AC (5.1‐ and 2.9‐fold for sIgG, and 3.6‐ and 5.0‐fold for sIgG_1_) comparing *t* = 3 to baseline (Figures 2B,C, S6). However, for BPE‐AC, this increase was already significant at the end of the up‐dosing phase (*t* = 2). Compared to placebo at *t* = 3, which hardly changed over time (between 0.6‐ and 1.1‐fold difference for all sIgG and sIgG_1_), the induction of BPE‐sIgG and sIgG_1_ reached significance in both active treatment groups.

In case of sIgG_4_, the fold increase (*t* = 1 to *t* = 3) was highly significant for BPE‐AC (13.9‐fold increase for BPE and 20.8‐fold for Bet v 1), and at *t* = 3, both BPE‐ and Bet v 1‐sIgG_4_ were significantly higher compared to placebo *t* = 3 (Figure 2D). For BM41 treatment, BPE‐ and Bet v 1‐sIgG_4_ at *t* = 3 were significantly higher than *t* = 1 and *t* = 2. However, compared to placebo, it did not reach significance, and the sIgG_4_ fold increases were 3‐fold lower compared to BPE‐AC. In BPE‐AC, the sIgG_4_/sIgG_1_ ratio significantly increased between *t* = 1 and *t* = 3 (3‐fold) and *t* = 2 and *t* = 3 (doubled), whereas for BM41 and placebo, it remained constant (Figure 2E), further confirming that BM41 is less potent in sIgG_4_ induction (Figure 2F).

Overall, we observed a divergence regarding the immune‐skewing effect induced by BM41 versus BPE‐AC. While the BM41 treatment resulted in an IgG_1_‐dominated sIgG response, BPE‐AC instead rather provoked an sIgG_4_ response.

### 3.4 BPE‐AC Induces Stronger Serum Inhibitory Activity Than BM41

**FIGURE 3:**
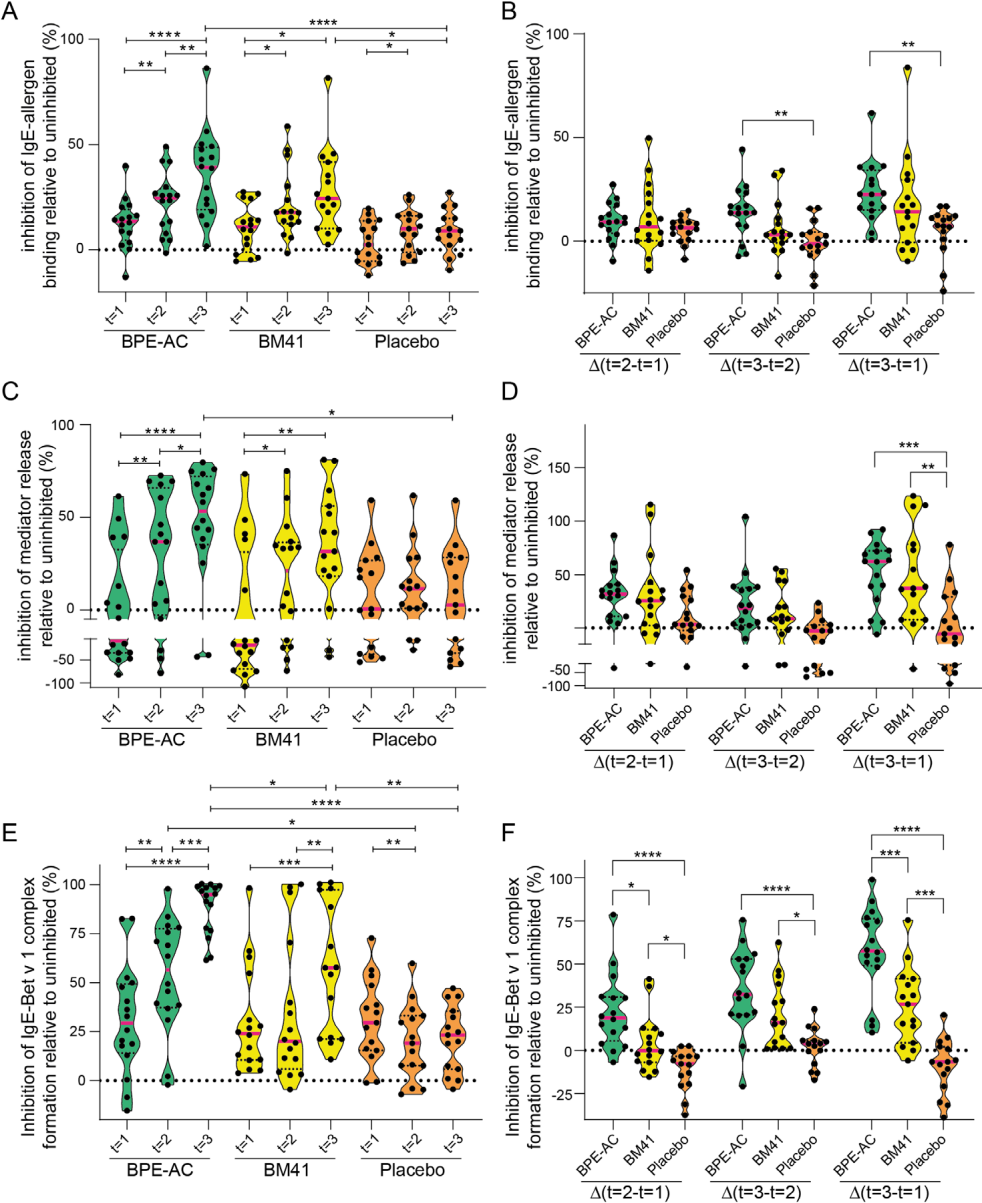
Serum inhibitory activity for Bet v 1‐specific IgE is induced in course of the active treatment with BM41 and BPE‐AC. The inhibition of IgE‐Bet v 1 binding was determined by inhibition ELISA (A). The placebo arm remained at a relatively constant level ranging between 2.5% and 9%. The inhibition of mediator release by patients' sera was investigated using a human reference serum containing high levels of Bet v 1‐specific IgE (C). FAB competition (E) assay was performed using serum from SCIT‐treated subjects. For placebo, the inhibition of complex formation remained at a constant level at around 20%–30%, reflecting at all time points the basal inhibition effect of BM41 and BPE‐AC patients' sera prior to treatment start, and around 0 in the Δ‐values (F). The data derived from all three assays were expressed as relative to the uninhibited reference (100%). Data are shown as violin plots with individual data (median is indicated by the red bar). The corresponding delta graphs (*t* = 2−*t* = 1, *t* = 3−*t* = 2, and *t* = 3−*t* = 1) are also shown (B, D and F). Of note, for all these approaches, IgE‐depleted patients' sera were used for the allergen preincubation step, and the reference IgE was derived from a human sera pool containing high Bet v 1‐sIgE to ensure experimental consistency across all samples. BPE‐AC, birch pollen extract‐based active comparator. *p ≤ 0.05; **p ≤ 0.01; ***p ≤ 0.001; ****p ≤ 0.0001.

The patients' sera were screened for inhibitory activity preventing sIgE‐Bet v 1 binding, complex formation, and IgE‐mediated basophil degranulation. In the inhibition ELISA, BM41 and the active comparator displayed a steady increase in inhibition of sIgE‐allergen binding throughout the treatment period (Figures 3A,B, S7A and S8). In BPE‐AC‐treated patients, the inhibition increased from 13.6% (*t* = 1, median of all patients) to 39.1% (*t* = 3, Δ22.7%), in BM41 from 10.9% to 24.5% (Δ14.3%), whereas placebo remained constant. For BPE‐AC, Δ*t* = 3−*t* = 1 and Δt = 3−*t* = 2 (Δ13.8%) were significant compared to placebo, whereas the Δ‐values of the BM41 group were not.

To assess if the BM41 treatment‐induced inhibitory effect of the patients' sera might also be sufficient to intervene in functional IgE‐allergen binding, we performed an iMRA (Figures 3C,D, S7B, S9, and S10). The treatment with BM41 induced an inhibition of mediator release of Δ37.2% from a median of −18.6% at *t* = 1 to 31.6% at *t* = 3, which was not as pronounced as the comparator BPE‐AC (Δ62.3%) but quite distinct from placebo (Δ‐5.5%). Only the BPE‐AC group was significant compared to placebo at *t* = 3. The Δ‐values for *t* = 3–*t* = 1 were significant for both active treatments compared to placebo.

In another approach to determine the serum inhibitory activity for sIgE induced throughout the treatments, we performed a competition (Figures 3E,F, S7C, S11 and S13) and inhibition FAB assay (Figures S7D–F and S12) to evaluate the suppression of CD23‐mediated sIgE‐Bet v 1 complex formation. In the FAB competition assay, the serum inhibitory efficacy increased from 29.3% (*t* = 1) to 56.5% (*t* = 2) and 95% (*t* = 3) in BPE‐AC (*t* = 1 to *t* = 3 Δ57.6%). Thus, the Δ*t* = 3−*t* = 1 of the BPE‐AC group was twice as high as that of the BM41 group (Δ26.9%), which increased from 24% (*t* = 1) to 57.7% (*t* = 3). The difference between both active treatment groups was significant at Δ*t* = 2−*t* = 1 and Δ*t* = 3−*t* = 1, indicating that BPE‐AC induces a quicker serum inhibitory activity. Noteworthy, all three Δ‐values of the BM41 group were significantly higher than those of the placebo group.

**FIGURE 4:**
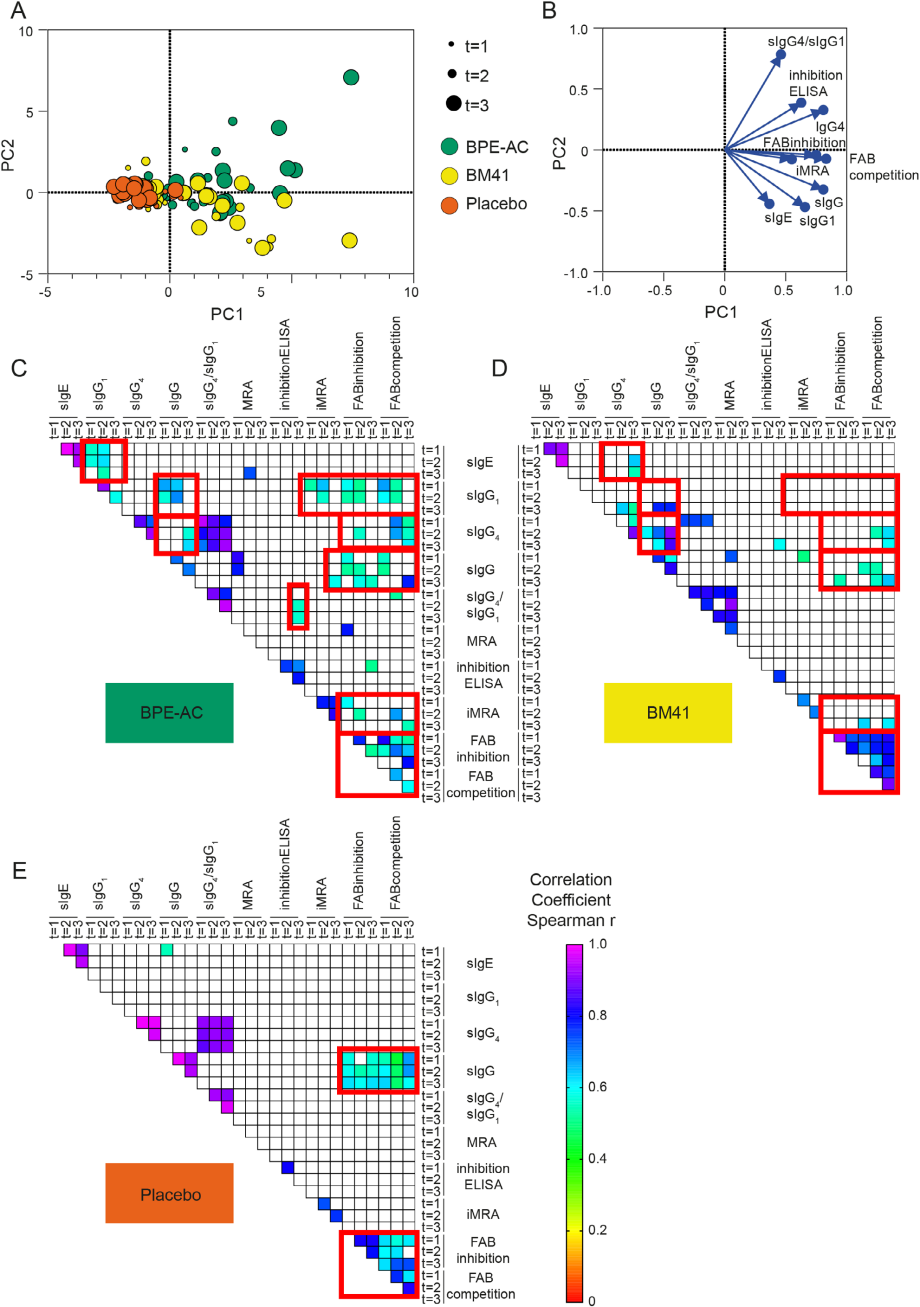
Distinct response and parameter correlation pattern in BPE‐AC‐ versus BM41‐treated patients. Bet v 1‐specific IgG, IgG_1_, IgG_4_ and IgE, the IgG_4_/IgG_1_ ratio, inhibition ELISA, FAB inhibition, FAB competition, and the iMRA were used to compute the PCA (A), with the corresponding loading graph (B). Only the two PCs with the largest eigenvalue were used for the graphical representation. Treatment specific patients' clusters were highlighted in the respective colors (BPE‐AC = green, BM41 = yellow, placebo = red). Correlation matrices (Spearman's rank correlation) of all three time points for BPE‐AC (C), BM41 (D) and placebo (E) are shown as heatmaps, only significant correlations are shown. In all three groups, the FAB inhibition correlated with FAB competition, and only the actively treated groups also correlated with the iMRA. In the BM41 group, a correlation between sIgE and sIgG_4_ (*r* = 0.54) at *t* = 3 was observed, which was not the case for BPE‐AC, most likely due to the higher, nonproportional induction of sIgG_4_ in BPE‐AC. In BM41, there was no correlation between sIgG_1_ and the inhibition assays, however, sIgG_4_ and sIgG correlated with FAB competition at *t* = 2 (*r* = 0.54 both) and *t* = 3 (*r* = 0.60 and *r* = 0.65, respectively). Red boxes highlight the results mentioned in the text. BPE‐AC, birch pollen extract‐based active comparator; FAB, facilitated allergen binding assay; iMRA, inhibition mediator release assay; MRA, mediator release assay; s, specific; PCA, principal component analysis.

By combining all investigated parameters in a principal component analysis (PCA), including Bet v 1‐specific immunoglobulins and data derived from inhibition ELISA, FAB inhibition, FAB competition, and the iMRA, a clear separation of the BPE‐AC group from placebo, which remained stationary at < PC1 at all time points, became evident over time (Figure 4A,B). Both active treatment groups increased in PC1 over time but clearly distributed across PC2 (BPE‐AC: PC2 > 0, BM41: PC2 < 0), which is mainly defined by sIgG_4_/sIgG_1_ ratio as indicated by the loading plot.

To investigate correlations among Bet v 1‐sIgE and sIgG titers and the serum inhibitory activity, Spearman's correlation coefficients were determined, and moderate correlations were defined as *r* = 0.40–0.69 and strong correlations as *r* > 0.70 (Figure 4C–E). In BPE‐AC, sIgG_1_ correlated with sIgE and sIgG at *t* = 1 (*r* = 0.56 and *r* = 0.67) and *t* = 2 (*r* = 0.62 and *r* = 0.69, respectively) but not at *t* = 3. At *t* = 3, there was a correlation between sIgG and sIgG_4_ (*r* = 0.63) and between inhibition ELISA and the sIgG_4_/sIgG_1_ ratio (*r* = 0.56). Regarding the serum inhibitory activity, the iMRA correlated with sIgG_1_ at *t* = 1 and *t* = 2 (*r* = 0.53 and *r* = 0.56, respectively) and with sIgG at *t* = 3 (*r* = 0.59). Similarly, the FAB competition correlated with sIgG_1_ at *t* = 1 and *t* = 2 (*r* = 0.67 and *r* = 0.53, respectively) and with sIgG_4_ at *t* = 2 and *t* = 3 (*r* = 0.67 and *r* = 0.61, respectively), while all time points correlated with sIgG. These data indicate that the serum inhibitory activity early in AIT is dominated by sIgG_1_ and later by sIgG_4_. In the BM41 group at *t* = 3, both sIgG1 and sIgG4 correlated with sIgG (*r* = 0.78 and *r* = 0.85, respectively), suggesting that both subclasses contributed to the composition of sIgG. In contrast, in BPE‐AC at *t* = 3, only sIgG_4_ correlated with sIgG (*r* = 0.63).

Overall, the PCA and correlation results suggest that there is an immune‐skewing effect toward Th1/IgG_1_ induced by the BM41 treatment, and toward a “modified Th2”/IgG_4_ response by BPE‐AC.

### 3.5 BM41‐Induced IgG_4_ Lacks Potential to Inhibit Mediator Release

**FIGURE 5:**
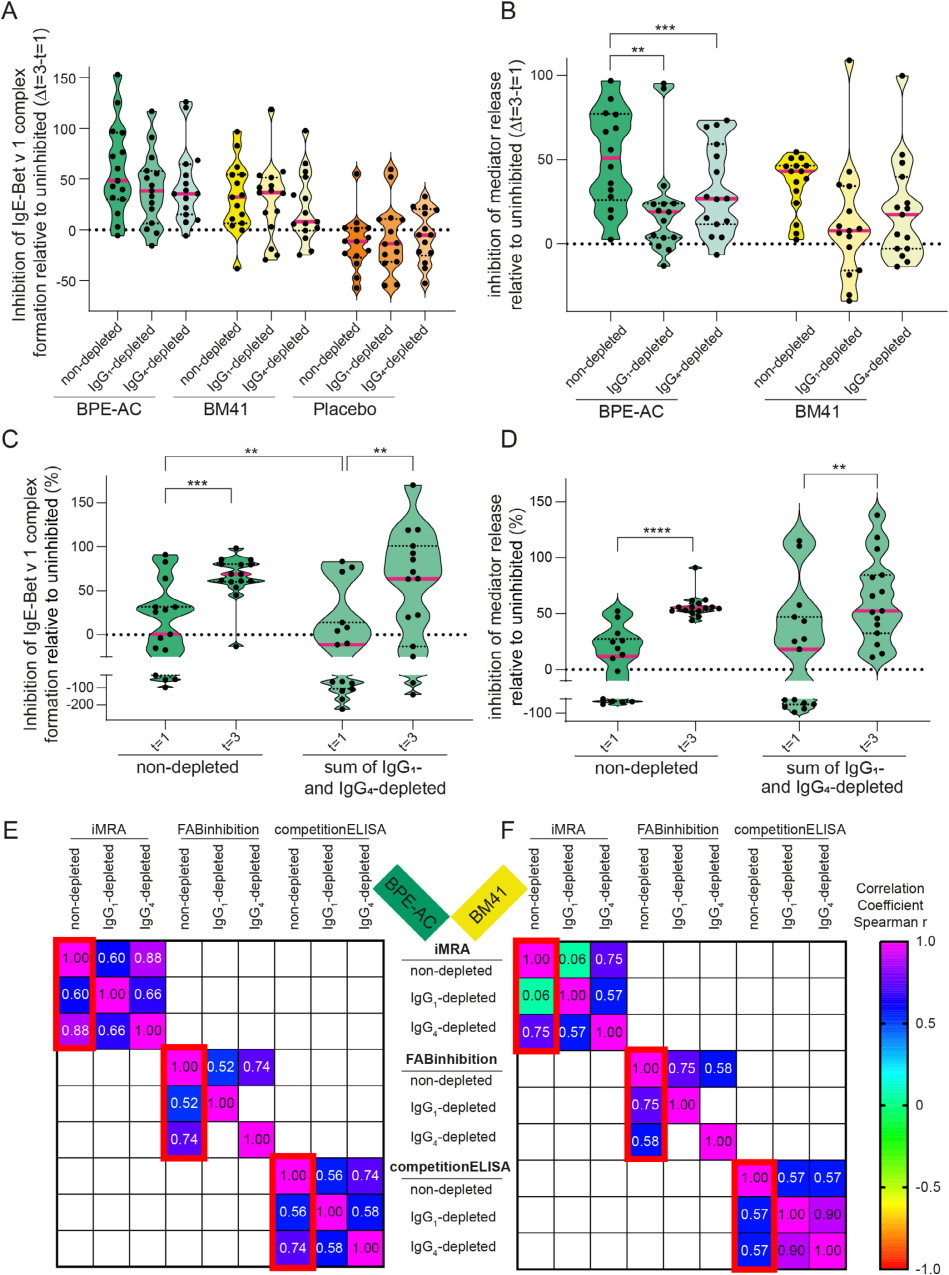
Serum inhibitory activity of IgG_1_‐ and IgG_4_‐depleted sera is reduced in BM41‐treated patients indicating a lack of functionality of the induced IgG antibodies. FAB inhibition assay was performed using the non‐, IgG_1_‐ and IgG_4_‐depleted sera (A). The depletion procedure resulted in a dilution of the samples of approximately 21%–28%. Since this dilution effect was not significant, we have decided not to correct for it. Inhibition of mediator release by depleted and nondepleted sera induced by Bet v 1‐cross‐linking of patients' serum IgE was investigated (B). The data derived from both assays were expressed as relative to the uninhibited reference (100%), the Δ*t* = 3−*t* = 1 was calculated and data are shown as violin plots with individual data (median is indicated by the red bar). For BPE‐AC, Δ*t* = 3−*t* = 1 showed 38.5% inhibition by IgG_1_‐depleted and 35.4% by IgG_4_‐depleted versus 48.5% by nondepleted control serum in the FAB inhibition assay, and 18.9% IgG_1_‐depleted, 26.8% IgG_4_‐depleted, and 50.9% by nondepleted control serum in iMRA. Regarding inhibition of complex formation (A) in BM41 group, 36.6% inhibition was achieved by IgG_1_‐depleted, 8.2% by IgG_4_‐depleted, and 32.2% by nondepleted control. Thus, IgG_1_‐depleted sera were like nondepleted placebo (−11.4%). In both assays, the sum of inhibition achieved by the IgG_1_‐ and the IgG_4_‐depleted sera nicely resembles the percentage inhibition obtained with the nondepleted sera, as exemplified with the BPE‐AC group (C‐D). Spearman's rank correlation matrix of the Δ*t* = 3−*t* = 1 values of the non‐, IgG_1_‐ and IgG_4_‐depleted sera of the BPE‐AC (E) and BM41 (F) group, only significant correlations are shown. Red boxes highlight the results mentioned in the text. BPE‐AC, birch pollen extract‐based active comparator; FAB, facilitated allergen binding assay; iMRA, inhibition mediator release assay; s, specific. **p ≤ 0.01; ***p ≤ 0.001; ****p ≤ 0.0001.

To assess which IgG subclass is responsible for the observed IgE‐blocking activity, the FAB inhibition assay and the iMRA were carried out with IgG_1_‐ and IgG_4_‐depleted patients' sera. The efficacy of IgG_1_‐ and IgG_4_‐depletion was confirmed by ELISA (Figures S14 and S15). In the case of BPE‐AC, Δ*t* = 3−*t* = 1 showed that both IgG subclasses contribute to a similar extent to the serum inhibitory activity (Figures 5A,B, S16 and S17A–D). In contrast, BM41‐induced sIgG_1_ contributed less to the inhibition of complex formation compared to wild‐type‐induced sIgG_1_, as the Δ*t* = 3−*t* = 1 of IgG_4_‐depleted sera was reduced by 24% compared to the nondepleted control. In iMRA, the IgG_1_‐depleted fraction (still containing IgG_4_) was reduced in the BM41 group (7.9% inhibition by IgG_1_‐depleted and 17.4% by IgG_4_‐depleted vs. 43.0% by nondepleted), which is in line with the lack of sIgG_4_ induction by the BM41 treatment. However, due to the high standard deviation, these differences were not significant. In both assays, summing up the serum inhibitory activity of both IgG classes, IgG_1_ and IgG_4_ (in average 52.4% inhibition at *t* = 3 for the iMRA and 63.5% for the FAB inhibition), resembled very well the activity achieved by the IgG nondepleted serum (55.0% for the iMRA and 68.7% for the FAB inhibition), as exemplified by the BPE‐AC group (Figure 5C,D). In a competition ELISA using Bet v 1‐sIgE antibodies, both IgG classes, IgG_1_ and IgG_4_, contributed equally to the IgE‐blocking activity in both active treatment groups (Figure S17E).

The correlation matrix of Δ*t* = 3−*t* = 1 of the data of all three inhibitory assays (Figure 5E,F) exposed that in the BPE‐AC group the results obtained using nondepleted sera correlated significantly with those of the IgG_4_‐depleted sera (*r* = 0.74–0.88) and less with IgG_1_‐depleted sera (*r* = 0.52–0.60). In the BM41 group, the correlation pattern shifted in favor of the IgG_1_‐depleted fractions in the case of the FAB inhibition and competition ELISA. However, the iMRA only correlated with the IgG_4_‐depleted sera (*r* = 0.75 vs. *r* = 0.06), suggesting that IgG_1_ dominated the serum inhibitory activity observed in this assay.

## 4 Discussion

Despite several clinical trials investigating the efficacy of hypoallergen‐based AIT vaccine candidates [3, 17, 18, 19], studies offering detailed characterization of the modulation of the allergen‐specific antibody response, regarding IgE‐mediated responses and the blocking thereof, during the first months of immunotherapy are scarce. In general, most well‐conducted studies lack an active comparator with confirmed efficacy, like a licensed pollen extract‐based AIT vaccine, necessary to put the findings into context to sufficiently assess their therapeutic potential [3, 17]. The treatment with Bet v 1 hypoallergenic candidates either provoked unexpected adverse side effects or showed no significant difference compared to an active comparator or even to placebo regarding the improvement of symptom scores [3, 7].

Here, we investigated safety (primary endpoint) and surrogate efficacy outcomes (secondary endpoints) of the Bet v 1‐fold‐variant BM41 in birch pollen‐allergic rhinoconjunctivitis patients. Reporting of hypoallergenicity, adverse events, and SPT was used to determine safety, and serum‐derived biomarkers were used to address AIT efficacy, including specific IgE, IgG, IgG_1_, and IgG_4_, basophil degranulation, facilitated antigen presentation, and serum inhibitory activity for IgE in respect of epitope‐masking in FcεRI‐ and CD23‐mediated experimental set‐ups.

We observed that BM41 is not as safe and effective as the conventional treatment with the licensed comparator. BM41 was hypoallergenic, considering that the response of serum IgE to BM41 at *t* = 1 was half of the response triggered by wild‐type Bet v 1, and that 6–7‐fold more BM41 was required in the SPT to achieve a similar wheal size as with Bet v 1. However, based on the preclinical in vitro observations showing a 30‐/55‐fold reduction in allergenicity (investigated by mediator release assays/basophil activation test), we expected a more pronounced in‐human hypoallergenicity [10]. However, most adverse events occurred in the BM41 group, which might be explained by an unforeseen imbalanced distribution of sIgE that occurred during randomization of the patients, with those in the BM41 group by trend having higher sIgE than the comparator groups. Therefore, we strongly recommend including sIgE as a randomization criterion in future AIT studies. Pauli et al. reported that treatment with recombinant Bet v 1 was accompanied by a larger number of local reactions (swelling, itching, and pain), comparable to the 50% of patients from the BM41 group suffering from immediate local reactions [8]. The occurrence of SAE due to subcutaneous administration of recombinant Bet v 1 or of structurally folded and unfolded Bet v 1 derivatives has been described previously [7, 8]. The administration of synthetic hypoallergenic Bet v 1 derivatives was also reported to result in systemic side effects. In particular, asthma, dyspnea, gastrointestinal reaction, and circulatory dysregulation only occurred in actively treated patients [7]. A significant induction of Bet v 1‐sIgE was further documented for subjects receiving the hypoallergenic vaccine candidate [17]. This raises two questions: Is clinical safety ascertained in all patients by a hypoallergenic product? And does the hypoallergenicity prior to the treatment endure during the treatment?

The secondary endpoint of our study was efficacy with emphasis on the modulation of allergen‐specific humoral immune responses, as a surrogate for clinical efficacy. Serum inhibitory activity was induced by both active treatments. Throughout the study, BM41 was less effective than BPE‐AC (Δ*t* = 3−*t* = 1 inhibition ELISA: 14.3% vs. 22.7%; iMRA: 37.2% vs. 62.3%; FAB competition/inhibition: 26.9/21.7% vs. 57.6/59.3%). Especially when comparing the second time point, which was obtained upon reaching the maintenance dose, it became evident that the treatment with BM41 was less effective, implying that BPE‐AC initiates a more rapid modulation of the beneficial humoral immune response (immune‐skewing to IgG_4_). Clinical efficacy is usually interpreted based either on in‐season symptom/medication scorings or provocation tests. Our study was planned as a proof‐of‐concept phase I/IIa trial and, thus, not powered for symptom/medication score analyses. The final time point in our study is comparable with data obtained by Würtzen et al., who investigated the therapeutic effects of birch SCIT with BPE‐AC eight to 10 months posttreatment and provided evidence for a treatment‐associated increase of non‐IgE serum inhibitory activity, which was accompanied by a reduction of seasonal symptoms and medication usage [20]. The authors observed a significant inhibition of IgE binding comparable to our inhibition ELISA data, a reduction of facilitated antigen presentation by 7%–78%, and a significant inhibition using the FAB inhibition assay ranging from 14% to 80% compared to placebo.

SCIT using birch pollen extracts and the treatment‐induced improvement of symptoms was studied extensively, and the general consensus is that the treatment is safe and efficacious [16, 21, 22, 23], whereas SCIT with recombinant wild‐type Bet v 1 was proven less effective compared to birch pollen extracts [8]. Several immunomodulatory properties have been described for pollen‐derived compounds [24, 25, 26, 27, 28, 29], which may largely be lost when only a single protein is administered, where the immunomodulation is mostly solely provided by the adjuvant of choice [30, 31].

Our findings that the fold variant induced less sIgG_4_, associated with a reduced serum inhibitory activity for IgE, were surprising since BM41 outperformed recombinant Bet v 1 in preclinical mouse models, where BM41 immunizations resulted in a Th1 skewing [10]. In this first‐in‐human study, we were able to reproduce this Th1‐skewing effect; however, contrary to our expectations, the BM41‐induced Th1/IgG1 response proved to be less efficient than the extract‐induced “modified Th2”/ IgG4 response. Since in our study all three arms received the same adjuvant and the active treatment groups the same dosage of (hypo)allergen [16], the alteration in immunogenicity is either resulting from pollen extract‐derived adjuvanticity or from the disruption of the intact fold or posttranslational modifications of the wild‐type allergen. A major limitation of our study is that recombinant wild‐type Bet v 1 was not included for comparison, making it difficult to determine the cause of the difference in efficacy. Regarding the question of whether fold alterations play a role in AIT efficacy, Klimek et al. found that a Bet v 1 folding variant obtained by using alkaline conditions throughout protein purification without modifying its primary amino acid sequence was as effective in reducing clinical symptoms and inducing sIgG_4_ as an extract‐based comparator [3, 32]. However, no placebo arm was included in the study, and the patients received in total a 5‐fold higher concentration of the fold variant than those receiving the natural allergen, making a direct comparison difficult. Of note, in comparison to this fold variant, in BM41 five amino acids were substituted by the corresponding amino acids of Mal d 1 [10, 33]. Since these amino acids constitute a major IgE epitope of Bet v 1, they likely also represent an important IgG_4_ binding site [34].

The class switching to IgG_4_ relies on the Th2 cytokines IL‐4 and IL‐13, questioning the role of Th1 in AIT efficacy [35]. Strobl et al. reported that early in birch pollen AIT, epitope masking is mainly mediated by sIgG_1_ until 18 months of treatment when IgG_4_ takes over [36]. In our data, at *t* = 3, both IgG subclasses induced by BPE‐AC contributed to the blocking effect similarly. Since IgG_4_ is becoming a more important blocking antibody at a later stage of AIT, BM41, which showed reduced efficiency in IgG_4_ induction, might even prove to be less efficient with extended treatment. Here, we provide evidence that “modified Th2”/IgG_4_‐skewing AIT vaccines are superior in inducing IgE blocking activity than those resulting in IgG_1_‐dominated responses.

## Author Contributions

L.A., devised and performed most experiments. L.A. and L.K.T. wrote the manuscript and created the figures. S.A.V., M.W., A.K., and H.W. conducted experiments. S.A.S. provided Bet v 1‐specific IgE monoclonal antibodies. E.A.B., S.S., L.H.B., and L.K.P. provided ImmunoCAP technology for sIgG_1_ measurement. N.N. performed correlation analysis and created figures. F.S. and A.N. provided the formulated GMP drug products. L.K.T., F.F., C.B.J., and R.R. designed and organized the clinical trial. L.A., L.J., F.F., and R.R. devised the experiments and interpreted data. R.R. coordinated the study. All authors read and approved the manuscript.

## Conflicts of Interest

F.S. was, and A.N. is, an employee of Biomay AG. The remaining authors declare that the research was conducted in the absence of any commercial or financial relationships that could be construed as potential conflicts of interest. Ronald van Ree received consulting fees and/or speaker fees from Angany Inc., HAL Allergy BV, Citeq BV, ThermoFisher Scientific, ALK Abello, Reacta Healthcare Ltd., Mission MightyMe, and The Protein Brewery and has stock options from Angany Inc.

## Supporting information


Appendix S1.

## Data Availability

The data that support the findings of this study are available from the corresponding author upon reasonable request.
