# Supplementary material for: Subcutaneous Allergen Immunotherapy With Hypoallergenic Bet v 1 Compared to Conventional Extract: Poorer Blocking Antibody Capacity Dominated by IgG1 Instead of IgG4
Source: Allergy. 2025 Jun 2;80(7):2018–30. doi: 10.1111/all.16606 (PMC12261870; doi:10.1111/all.16606)
Supplement: Supplementary file 1 — Appendix S1. [file ALL-80-2018-s001.pdf]

SCIT with hypoallergenic Bet v 1 compared to conventional extract: poorer blocking antibody capacity dominated by IgG_1 instead of IgG_4

Lorenz Aglas[1,2◊*], Line Kring Tannert[3◊], Serge A. Versteeg[4], Scott A. Smith[5], Ewa A. Bartko[6], Mario Wenger[2], Amin Kraiem[2], Hannah Widauer[2], Natália Nunes[2], Sibile Sinkunaite[6], Frank Stolz[7], Laurian Jongejan[4], Angela Neubauer[7], Lars H. Blom[6], Fatima Ferreira[2], Lars K. Poulsen[6,8], Carsten Bindslev-Jensen[3,9], Ronald van Ree[4,10]

[1]Institute of Pathophysiology and Allergy Research, Center for Pathophysiology, Infectiology and Immunology, Medical University of Vienna, Vienna, Austria.

[2]Department of Biosciences and Medical Biology, University of Salzburg, Austria

[3]Odense Research Center for Anaphylaxis, Odense University Hospital, Odense, Denmark

[4]Department of Experimental Immunology, Amsterdam University Medical Centers, location AMC, Amsterdam, The Netherlands

[5]Department of Medicine, Vanderbilt University Medical Center, Nashville, TN, United States

[6]Department of Dermatology and Allergy, Allergy Clinic, Copenhagen University Hospital-Herlev and Gentofte, Copenhagen, Denmark

[7]Biomay AG, Vienna Competence Center, Vienna, Austria

[8]Department of Clinical Medicine, University of Copenhagen, Copenhagen, Denmark

[9]Department of Dermatology and Allergy Center, Odense University Hospital, Odense, Denmark

[10]Department of Otorhinolaryngology, Amsterdam University Medical Centers, location AMC, Amsterdam, The Netherlands

[◊] Lorenz Aglas and Line Kring Tannert contributed equally to this work.

*** Correspondence:**
Dr. Lorenz Aglas
ORCID ID: 0000-0002-1236-5934
Department of Biosciences and Medical Biology, University of Salzburg
Hellbrunner Str. 34, A-5020 Salzburg, Austria
Tel.: +43-662-8044-5819
Email: lorenz.aglas@plus.ac.at

Supplemental Material and Methods

Subjects and clinical trial information

Alutard SQ (BPE-AC) was given as a cluster up-dosing with 13 injections and 3 maintenance injections. Participants randomized to BM41 received 7 up-dosing injections and 4 maintenance doses. Placebo injections were given corresponding to BM41. The concentration of BM41 was defined based on the combination of commonly used major allergen doses in extracts and restricted by pre-clinical toxicity studies [1]. An example of a regular BPE-AC cluster up-dosing schedule compared to the BM41 up-dosing schedule is provided in Fig 1C. Details on baseline data at inclusion of the patients as well as the defined inclusion and exclusion criteria can be found in Tables S1, S2 and S3. None of the participants received AIT for any allergen at least 5 years prior to the study. Comorbidities (not specifically mentioned in the exclusion criteria) were not documented. All participants completed the clinical trial, except of one patient of the BM41 group that was withdrawn from the study due to a severe adverse event (SAE) occurring at visit 7. Sera derived from this patient collected at the first two time points were subjected to further analysis. BM41 and placebo samples were blinded prior to the analysis, whereas BPE-AC samples were not blinded. One patient (BPE-AC) did not meet the inclusion criteria (lower IgE levels), but still was included for further analysis due to ethical considerations.

Recombinant proteins

BM41 (formerly called BM4) was produced recombinantly and endotoxin-free under good manufacturing practice (GMP) conditions based on the described protocol (Biomay AG, Vienna, Austria) [2]. Both BM41 and placebo were formulated using aluminum hydroxide (Alhydrogel 2%, Brenntag, Vantaa, Finland) [1-3]. In immunological assays, recombinant Bet v 1.0101 was used (called Bet v 1 in the main text) [4]. Recombinant Bet v 1.0101 was expressed, purified and physico-chemically characterized as previously described [4]. The level of endotoxin contamination was measured as <0.3 ng/ml.

Reporting of adverse events

All symptoms occurring during or between hospital visits were recorded according to the Medical Dictionary for Regulatory Activities (MedDRA) and relationship to IMP and severity was assessed. Patients receiving BM41 or placebo were observed for immediate allergic reactions in the clinic two hours after each injection, and patients receiving BPE-AC were observed 30 minutes after each injection according to recommendations. Adverse events occurring between hospital visits were self-reported.

Skin-prick-tests

Skin-prick-test with common inhaled allergens was performed at inclusion. A titrated skin-prick-test (TSPT) was conducted with BM41 (diluted in saline solution, 0.9% NaCl) at t=1, t=2 and t=3. BM41 SPT solution (100 µg/ml) was titrated from 100% to 0.78% and was applied on the volar forearm in duplicates. At inclusion, a SPT with a single concentration of a birch pollen extract SPT solution (*Betula verrucosa* (108) 10 HEP, ALK, Denmark) was used. Histamine and a saline buffer solution were used as positive and negative controls, respectively (ALK,

Denmark). Wheal sizes were read after 15 minutes post pricking. The total area under the curve (AUC) was calculated for the dose-response curves of TSPT.

Immunoglobulin measurements in serum

Birch pollen extract (BPE)-, Bet v 1-, Bet v 2- and BM41-specific IgE, IgG_4 and IgG were measured in patients' sera by ImmunoCAP (Thermo Fisher Scientific, Uppsala, Sweden) according to the manufacturer´s instructions. For the first three, commercially available ImmunoCAPs were used (t3, t215 and t216, respectively), whereas BM41 was biotinylated and bound to streptavidin ImmunoCAPs (Ro212) at 1 µg per test, following the manufacturer's protocol.

IgG_1 measurements in serum with ImmunoCAP

IgG_1 was measured using an in-house assay based on the ImmunoCAP principle (ThermoFisher, Uppsala, Sweden). In brief, 40 µl of serum (diluted 1:100) was incubated either with commercially available CAPs for BPE (t3) or Bet v 1 (t215), or custom-made BM41-CAP. Allergen $sIgG_1$ was detected with β-galactosidase-labeled anti-IgG_1 (M1325 - anti-IgG1(CH2) monoclonal MoHu 161-1, Sanquin, Netherlands). The concentration of $sIgG_1$ was calculated using the sIgG calibration curve (ThermoFisher, Uppsala, Sweden) diluted three times and corrected for IgG_1, constituting 60% of all the combined four IgG subclasses. The cut-off value, 0.00121 ug/mL, was defined as the mean of the lowest point of the calibration curve, which was corrected for 8% of the mean, corresponding to 2xCV of the diluent.

Assessment of IgE-mediated responses

For the assessment of IgE-mediated responses the mediator release assay (MRA) and IgE-facilitated allergen binding (FAB) assay were used, see details below.

Serum inhibitory activity

Inhibition enzyme-linked immunosorbent assay (ELISA), inhibition MRA (iMRA), and FABinhibition/FABcompetition assays using IgE-depleted patients' sera were used to assess the serum inhibitory activity, see experimental details below.

Mediator release assay (MRA)

To assess the human high-affinity IgE receptor- (FcεRI-)mediated basophil degranulation facilitated by patients' IgE-allergen crosslinking, an MRA was performed. Rat basophil leukemia cells (RBL-2H3), transfected with FcεRI, were passively sensitized with patients' sera one day prior to the experiment. The assay was performed as previously reported [4]. Degranulation was triggered by Bet v 1 in concentrations ranging from 100 ng/ml to 0.1 pg/ml. Potential toxicity of human sera was assessed by using the colorimetric cell viability substrate MTT (3-(4,5-Dimethylthiazol-2-yl)-2,5-Diphenyltetrazolium Bromide, Sigma-Aldrich, Inc., St. Louis, MO, USA, Fig S3B, C). The percentage of mediator release for each human serum was expressed relative to the maximal release induced by cell lysis using Triton X-100 (Sigma-Aldrich, Inc.). After baseline subtraction (no antigen control for each serum), positive response curves (Fig S3A) were used to calculate the AUC for simplified representation (baseline=-5%).

Responding patients' sera were defined based on their general degranulation curve behavior and on the maximal release induced by the allergen (>0.5%).

For the inhibition MRA (iMRA) set-up, a human reference serum was used for sensitizing the RBL-2H3 cells with IgE. The patients' sera were IgE-depleted by heat inactivation for 1 hour (h) at 56°C. IgE-depletion efficacy was confirmed by ELISA (data not shown). Depleted patients' sera were pre-incubated with P3X63Ag8.653 cells (ATCC, Wesel, Germany) at 37°C for 1h for deactivation of the complement system, and incubated with either 0, 1, 10 or 100 ng/ml of Bet v 1 diluted in 1x Tyrode´s buffer (Sigma-Aldrich, Inc.) for 1h at 37°C before applying to the cells. Subsequent steps were performed according to the standard MRA protocol [4]. After subtraction of the control (no antigen), the AUC was calculated for each patients' serum for 1-100 ng/ml Bet v 1, expressed as relative to the uninhibited reference serum and in percentage inhibition. An example of three patients' sera (one for each treatment group) is provided in the supplemental material (Fig S9A). For the inhibition MRA (iMRA) using IgG_1- and IgG_4-depleted sera, 20 µl of each serum was used and IgE-depleted prior to the experiment.

IgE-facilitated allergen binding (FAB) assay

We performed FAB assays to monitor the ability of patients' serum IgE to bind to Bet v 1 and, consequently, resulting in the formation of Bet v 1-IgE complexes immobilized on B cells via CD23 interaction, the low IgE-binding receptor (also called FcεRII). For this purpose, we adapted the protocol published by Shamji et al [1, 5]. In short, a total volume of 50 µl of patients' serum was incubated with 100 ng/ml Bet v 1 for 1h at 37°C, complemented with $1x10^5$ Epstein–Barr virus (EBV)-transformed B cells and incubated for another hour at 4°C. Cells were stained with PE-labeled anti-human CD23 (BD Biosciences, San Jose, CA, USA), FITC-labeled anti-human IgE (KPL/medac GmbH, Wedel, Germany) and SYTOX™ Red Dead Cell Stain (Thermo Fisher Scientific, Waltham, MA, USA) for live/dead discrimination in the dilutions 1:40, 1:100 and 1:100, respectively. For analysis, a Cytoflex S (Beckman Coulter, Brea, CA, USA) and FlowJo v 10 software (FlowJo, LLC, Ashland, OR, USA) were used. The limit of quantification (LOQ) was used as threshold to distinguish sera that responded in the assay from non-responding samples. The LOQ was defined as the sum of the mean plus the 10-fold standard deviation of the negative control, which consisted of similarly treated EBV-transformed B cells in the absence of human IgE.

For the inhibition approach (FABinhibition), 10 ng/ml Bet v 1 were pre-incubated for 1h at 37°C with 1 µl of IgE-depleted patients' serum (as mentioned above). To facilitate IgE-Bet v 1 complex formation, 1 µl of an IgE-non-depleted human reference serum was added and incubated for another hour at 37°C, before addition of the EBV-transformed B cells. The reference serum contained a high level of Bet v 1-specific IgE antibodies, as determined by ImmunoCAP assays. The FABinhibition assay with the IgG_1- and IgG_4-depleted sera was performed with 5 µl of each serum (depleted or non-depleted, description of depletion protocol can be found below), 5 µl of 0.1 µg/ml Bet v 1 and 15 µl of a different human reference serum pool as mentioned above. In the competition set-up (FABcompetition), IgE-depleted patients' serum and reference serum were added simultaneously to the allergen and incubated for 1h at 37°C. All FAB experiments were performed in technical duplicates. Gating on single viable

CD23[+] anti-IgE[+] double-positive cells was performed as previously described [1]. The data were expressed as relative to the uninhibited reference serum (100% signal) and as a percentage of inhibition (inhibition=100 minus relative IgE-FAB value). Definition of ideal experimental conditions and compensation were conducted prior to the experiments [1].

Inhibition and competition enzyme-linked immunosorbent assay (ELISA)

The inhibition ELISA was performed as a slightly adapted version of the previously published protocol [1]. In short, 50 µl of Bet v 1 in a final concentration of 2 µg/ml were coated onto a Nunc MaxiSorp® flat-bottom 96 well plate (Thermo Scientific, USA) and after blocking with 0.5% bovine serum albumin incubated with IgE-depleted patients' sera (as mentioned above) in a dilution of 1:2 overnight at 4°C. This inhibition step followed a 2h incubation at room temperature with a human reference serum pool derived from five birch allergic patients in a dilution of 1:8. As detection antibody, an alkaline phosphatase-conjugated mouse anti-human IgE antibody (clone B3102E8, SouthernBiotech, Birmingham, USA) was used in a dilution of 1:1000. The absorbance of the colorimetric alkaline phosphatase substrate p-nitrophenyl phosphate (Sigma-Aldrich, St. Luis, MI, USA) was recorded at 405 nm after 3h. For data normalization, each absorbance value was expressed relative to the uninhibited reference serum pool (100%) and the percentage of inhibition was calculated thereof (inhibition=100 minus relative absorbance value). The assay was performed in technical duplicates.

The competition ELISA was performed with 0.001 µg/ml of two Bet v 1-specific human IgE monoclonal antibodies (clone: 23H5 and 3C10, produced as previously described [6]) recognizing different epitopes on Bet v 1 that were added simultaneously with the non-, IgG_1- and IgG_4-depleted sera to the Bet v 1 coated plate in a final dilution of 1:4 and incubated for 2h at RT. For detection a HRP-conjugated mouse anti-human IgE antibody (clone B3102E8, Southern Biotech, Birmingham, USA) diluted 1:2000 was used. The HRP substrate SureBlue TMB (3,3′,5,5′-Tetramethylbenzidine) Microwell peroxidase substrate (KPL, Gaithersburg, MD, United States) was used and plates were measured at a wavelength of 450 nm in a plate reader. The assay was performed in technical duplicates.

Depletion of IgG_1 and IgG_4 antibodies

Serum samples (300 µl) were depleted of IgG_1 and IgG_4 using either CaptureSelect IgG_1 or CaptureSelect IgG_4 human affinity matrices (100 µl, Thermo Fisher Scientific), respectively, as previously reported [7]. In short, affinity resins were incubated with serum for 30 min at room temperature (RT) and then over night at 4°C under constant rotation. Depleted serum supernatants were collected by centrifugation (1 min at 10,000 g) used in another depletion cycle using fresh affinity resins. For sufficient depletion, three depletion cycles were performed in total. Proof of efficacy of subclass depletion and determination of a potential dilution effect by the depletion procedure were performed by ELISA. Bet_v_1 (2 µg/ml) was coated on Nunc MaxiSorp® flat-bottom 96 well plates (Thermo Scientific, USA). Depleted and non-depleted sera were diluted 1:4 and a mouse anti-human IgG1-Fc HRP antibody (clone HP6001, SouthernBiotech, diluted 1:4000) and a mouse anti-human IgG4-Fc HRP (clone HP6025, SouthernBiotech, diluted 1:8000) were used as detection antibodies.

Statistical analysis

In Fig S6A-E, a paired t-test or 2-way ANOVA were performed for analysis within the same or different treatment groups, respectively. For those correlations where non-normally distributed data were included, the two-tailed Spearman´s rank correlation was used (in correlation matrices Fig 4C-E and Fig 5E-F), because Spearman´s rank correlation can be applied on both normal and non-normal data distributions effectively. For this, the absolute values of the Bet v 1-specific immunoglobulin data, the AUC values of the MRA, or the inhibition values relative to uninhibited in case of the inhibition ELISA, FABinhibition, FABcompetition, and the iMRA were used. Only statistically significant correlations ($p \leq 0.05$) are depicted in the correlation matrices. For normally distributed data (Fig S2C-E, S3E, S4D-E, S8B-C, S9D, S10, S11C-D, S12B, and S13B), a two-tailed Pearson correlation coefficient was computed using log-transformed data of Bet v 1-specific IgE and IgG_4 levels (Y = logY). Principal component analysis (PCA) was used to reduce the complexity of the analyzed parameters representative for serum inhibitory activity, including Bet v 1-specific IgG, IgG_1, IgG_4 and IgE, the IgG_4/IgG_1 ratio, inhibition ELISA, FABinhibition, FABcompetition, and the iMRA. The standard MRA and FAB were not included since not all patients responded in the assays. The data were scaled to have a mean of zero and standard deviation of one. The two PCs with the largest eigenvalue (PC1=4.03 and PC2=1.40) were used for the graphical representation, together representing 60.36% of the data variance (Fig 6 and S8). All statistical tests, including Pearson correlation, AUC calculation and PCA, were conducted using GraphPad Prism version 9.0 (GraphPad Software, San Diego, CA, USA). For the correlation matrices, R Studio and the package ggplot2 3.2.0 were used (R Studio Inc., Boston, MA, United States, version 4.2.1).

Supplementary Tables

Table S1. Demographic data and clinical response at inclusion

		**BPE-AC n=16**	**BM41 n=16**	**Placebo n=15**
Gender	M/F	8/8	8/8	9/6
Age	Mean ± SD	38.27 ± 15.20	32.94 ± 12.22	32.07 ± 12.08
	Range	22-65	20-63	20-54
Specific IgE (kU/L)				
BPE	Mean ± SD	11.33 ± 14.82	25.32 ± 33.02	16.28 ± 9.60
	Range	0.37-57.80	0.99-100.00	1.67-31.00
Bet v 1	Mean ± SD	8.93 ± 11.07	36.89 ± 77.47	14.23 ± 9.90
	Range	0.35-43.80	0.96-312.00	1.00-38.10
Duration of symptoms (years)	Mean ± SD	22.33 ± 14.46	19.73 ± 6.15	22.12 ± 11.79
	Range	3.75-59.77	8.80-27.82	2.80-50.75
Asthma	n (%)	2 (13%)	0 (0%)	3 (20%)
Atopic dermatitis	n (%)	1 (6%)	5 (31%)	2 (13%)
Other allergies				
Grass	n (%)	8 (50%)	12 (75%)	11 (73%)
House dust mites	n (%)	1 (6%)	3 (19%)	0 (0%)
Animals (dog, cat, horse)	n (%)	2 (13%)	3 (19%)	3 (20%)
Ragweed	n (%)	1 (6%)	2 (13%)	0 (0%)
Molds	n (%)	1 (6%)	0 (0%)	0 (0%)

Table S2. Clinical trial inclusion criteria

No.	Criteria
1	Signed informed consent
2	Age ≥18 ≤ 65 years
3	Moderate to severe birch-pollen-induced AR/ARC of at least 2 years according to the *Allergic Rhinitis and its Impact on Asthma (ARIA) guidelines* (Appendix 1, see the manual of procedures) with or without concomitant mild to moderate persistent asthma
4	FEV1>70% for patients with a history of asthma, FEV1>70% or PEF>80% for patients without a history of asthma
5	A positive SPT (mean wheal diameter ≥ 3mm compared to negative control and negative control should be negative) for birch pollen assessed within 1 year before randomization
6	Specific IgE against birch pollen extract ≥ 0.7 kU/L and against Bet v 1 ≥ 0.35 kU/L as determined by ImmunoCAP

Table S3. Clinical trial exclusion criteria

No.	Criteria
1	Chronic asthma with an FEV1<70 % of predicted value.
2	History of AIT (SCIT or SLIT) with birch pollen or tree pollen mix including birch pollen within the past 5 years
3	Ongoing AIT (SCIT or SLIT) with any allergen(s) during the study period
4	Vaccination within one week before or during the treatment phase.
5	Immunosuppressive or biological medication (e.g. IL-5, anti-IgE therapy) within the last six months prior to inclusion and up to end of trial (EoT).
6	Severe immune disorders (including auto-immune diseases) and/or diseases requiring immunosuppressive drugs.
7	Uncontrolled asthma or other active respiratory diseases
8	Active malignancies or any malignant disease during the previous 5 years.
9	Severe uncontrolled diseases that could increase the risk for patients participating in the study, including but not limited to: cardiovascular insufficiency, any severe or unstable lung diseases, endocrine diseases, clinically significant renal or hepatic diseases, or haematological disorders.
10	Renal insufficiency
11	Active inflammation or infection of the target organs (nose, eyes or lower airways) at the start of the study.
12	Diseases with a contraindication for the use of adrenaline (e.g. hyperthyroidism, glaucoma).
13	Use of systemic steroids within 4 weeks before start of the study and during the study.
14	Treatment with systemic and local β-blockers.
15	Known allergy towards constituents of the vaccine
16	Pregnancy, lactation or inadequate contraceptive measures for women of child-bearing age (adequate contraceptive measures will beintrauterine device or hormonal contraception (birth control pill, implant, transdermal patch, vaginal ring or depot injection). It is also accepted, if the female patient is permanently sterile or infertile, if her sole partner is permanently sterile, or if they use both condom and diaphragm, The definition of sterile or infertile is surgically sterilized (vasectomy/bilateral salpingectomy, hysterectomy and/or bilateral ovariectomy) or post menopause defined as a non-menstrual period of at least 12 months before inclusion in the study.
17	Alcohol, drug or medication abuse within the past year.
18	Any clinically significant abnormal laboratory parameter at screening.
19	Lack of cooperation or compliance.
20	Any physical or mental condition that precludes administration of SCIT, compliance or participation in a clinical trial.
21	Patients who are students or employees of the institution or 1st grade relatives or partners of the investigators
22	Participation in a clinical trial within 3 months prior to the current trial.

Supplementary Figures

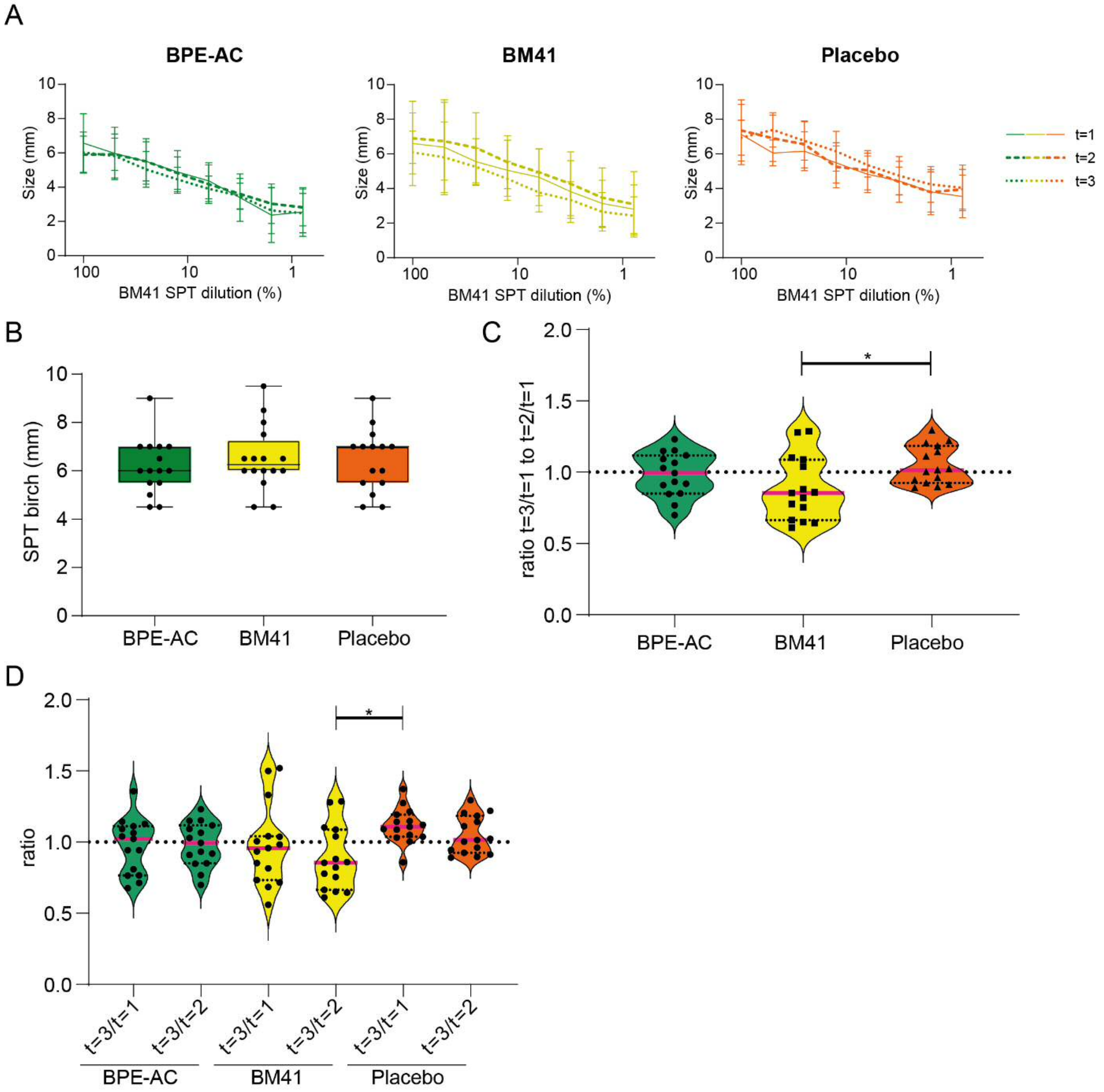

Figure S1 The reactivity of the patients to BM41 was investigated using a TSPT at the three various time points of the treatment (A). A SPT using a birch pollen extract solution was performed at the inclusion phase of the patients' screening (B). Ratios of AUC of TSPT with BM41 between the different time points (C, D). Total number of patients was n=15 per group. Of note, the borderline patient in BPE-AC as well as the patient in the BM41 group, who was withdrawn, were not included in the analysis of the SPT. This also refers to Fig.1G in the main text. BPE-AC, birch pollen extract-based active comparator; SPT, skin-prick-test. *$p \leq 0.05$.

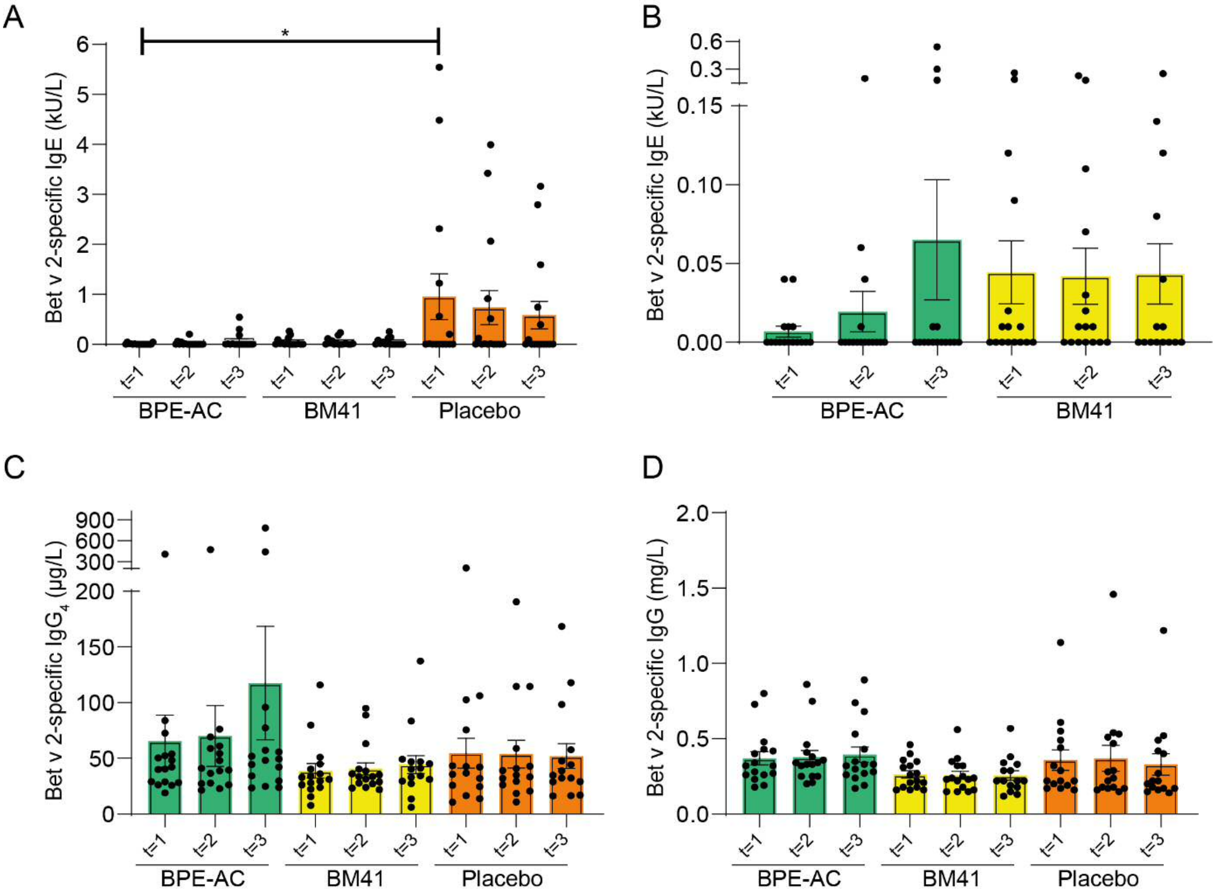

Figure S2 Bet v 2-specific IgE (A, B), IgG_4 (C) and IgG levels (D) in patients' sera collected at the three different time points of the study were determined with ISAC assay. The treatment with BPE-AC resulted in a negligible (<0.5 kU/L) Bet v 2 de-novo sensitization in 3 patients. BPE-AC, birch pollen extract-based active comparator. *$p \leq 0.05$.

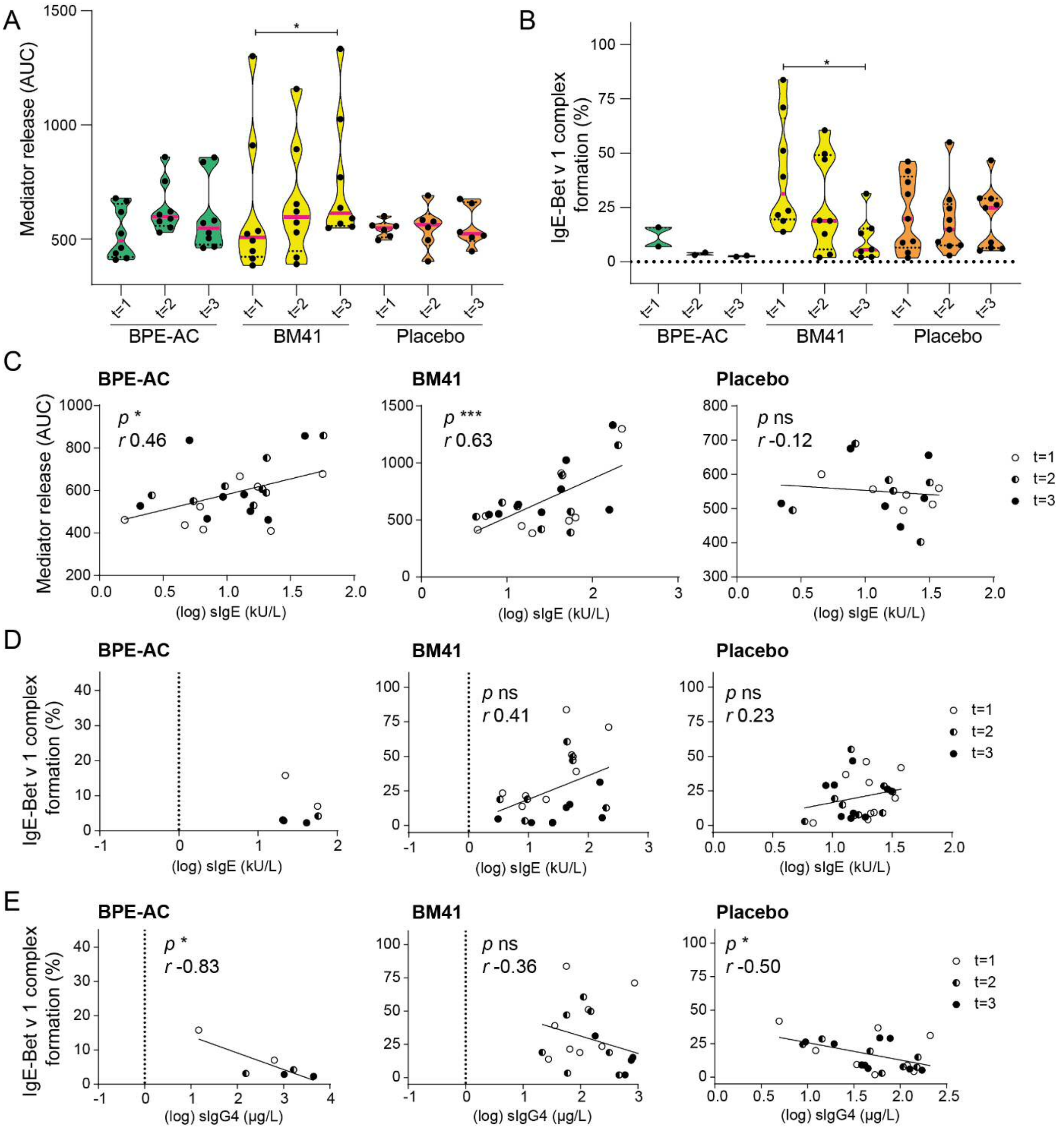

Figure S3 Assessment of the functionality of Bet v 1-specific IgE in course of the treatment with BM41 compared to BPE-AC and placebo. BM41 significantly induced mediator release, as investigated by the MRA (A), but reduced IgE-Bet v 1 complex formation (B). In the MRA, out of 47 patients' sera, 22 (n=8 BPE-AC, n=8 BM41, and n=6 placebo) triggered significant degranulation upon allergen crosslinking. The AUC for the full dose-response curves were calculated for each group and time point, and revealed that BM41 treatment resulted in a significant elevation of mediator release between t=1 and t=3, respectively (p = 0.0112). In the BPE-AC and the BM41 group, the mediator release correlated well with the Bet v 1-specific IgE levels (r = 0.46, p = 0.24 and r = 0.63, p = 0.0009, respectively). Regarding the CD23-mediated response (B), for only 19 out of 47 patients' sera (n=2 BPE-AC, n=8 BM41, and n=9 placebo) a positive signal for IgE-Bet v 1 complex formation was obtained exceeding the LOQ, reflecting the difference in sIgE titers at inclusion in both groups. In contrast to the MRA, in the FAB assays the contribution of sIgGs to the outcome of the assay has to be considered, thus, the percentage of IgE-Bet v 1 complex formation correlated rather with the

$sIgG_4$ than with sIgE titers. Of note, the main difference between FAB and mediator release assay is that in case of the latter only IgE binds to FcεRI expressed on the cells, while other immunoglobulins are washed away before antigen stimulation, meaning that IgG is not contributing to the outcome of the MRA and only the potency of IgE is investigated. In both assays, high sIgE levels (> approx. 17.6 kU/L, CAP class 5-6) are required, thus, only 6-8 patients per group responded in the MRA and 2-9 in the FAB. Data are shown as violin plots with individual data (median is indicated by the red bar). Pearson correlation was performed with MRA (C) and FAB assay data (D) and transformed Bet v 1-specific IgE values (Y=Log[Y], C and D) or $sIgG_4$ (E) per responding patient. AUC, area-under-the-curve; BPE-AC, birch pollen extract-based active comparator; FAB, facilitated allergen binding; MRA, mediator release assay. *$p \leq 0.05$; ***$p \leq 0.001$.

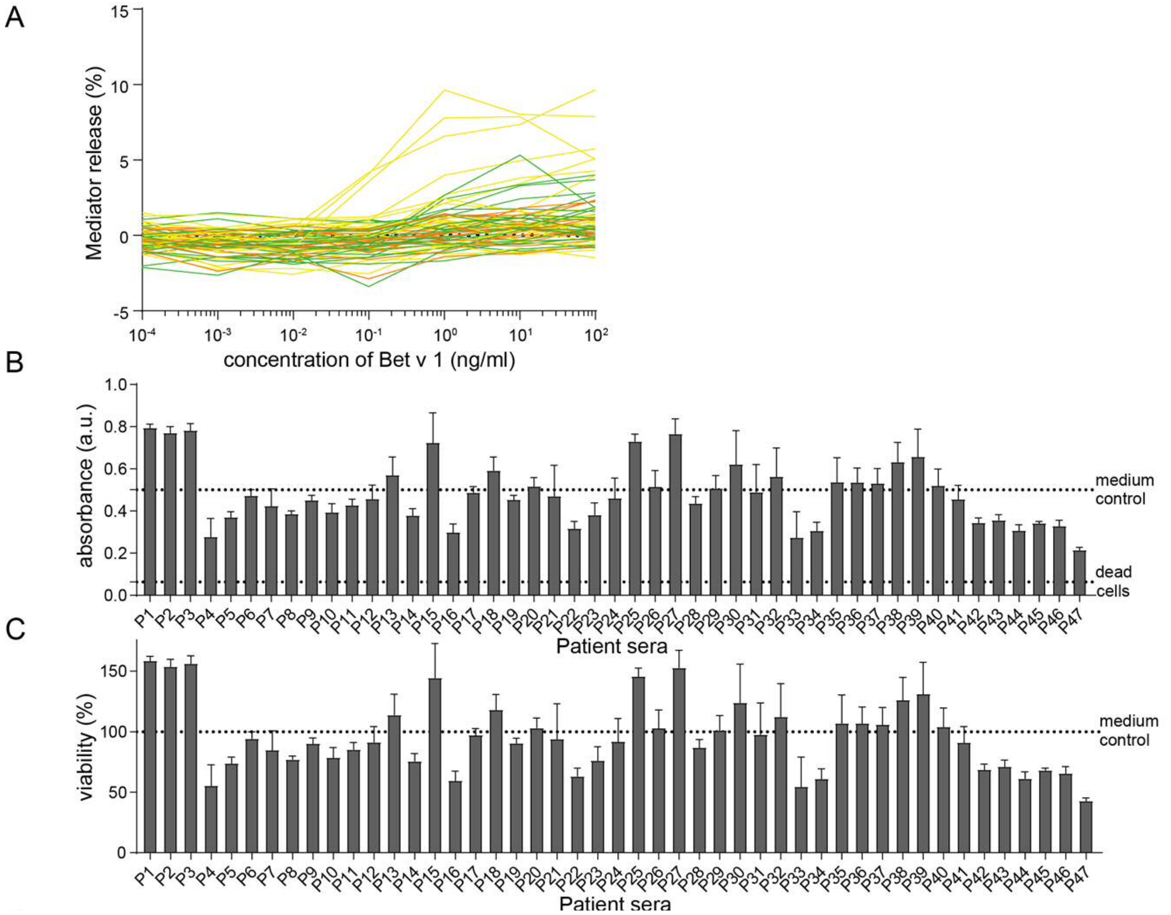

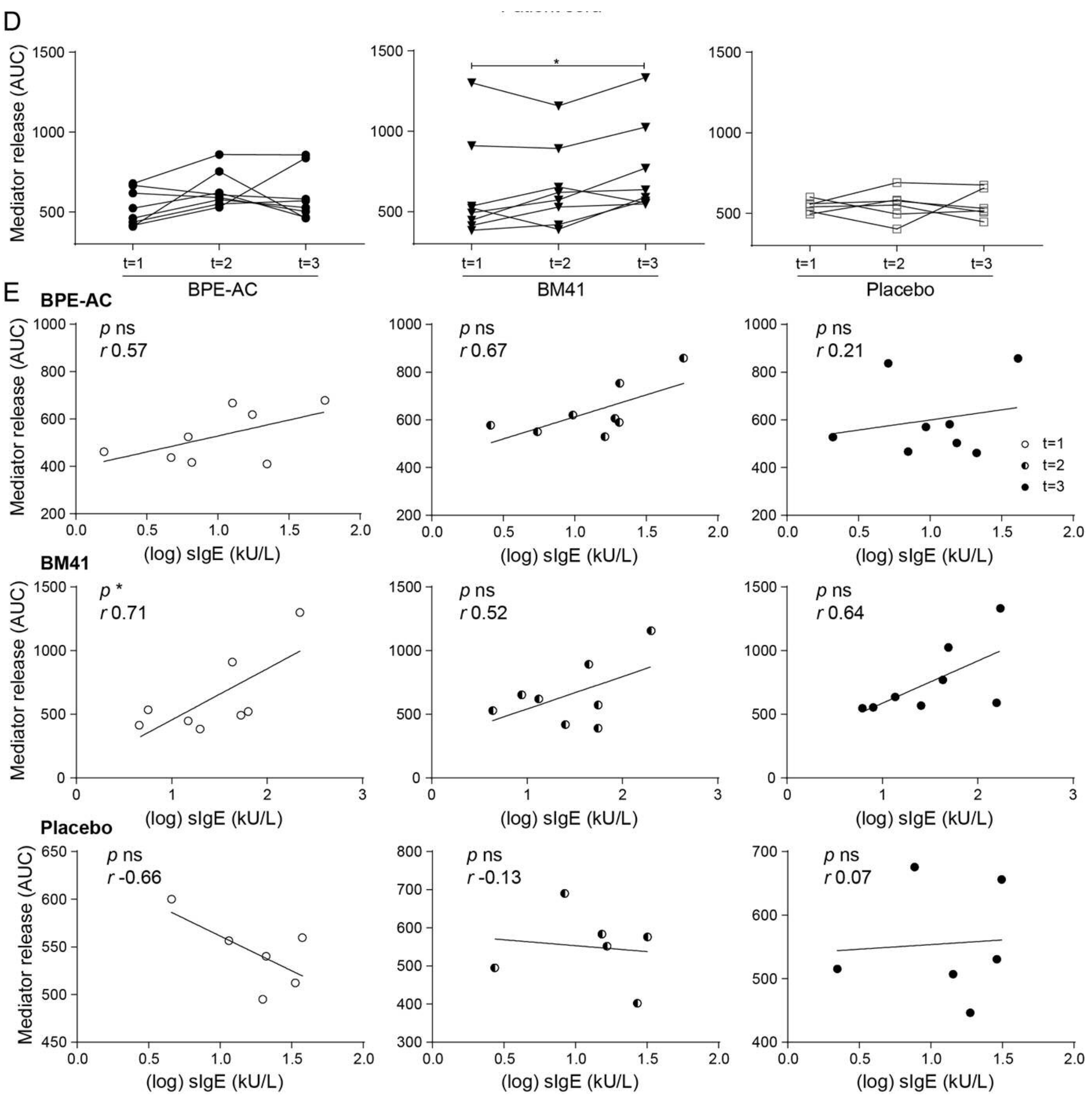

Figure S4 Positive mediator release dose-response curves of cells sensitized with patients' serum and triggered by 100 ng/ml to 0.1 pg/ml of Bet v 1 (A). Responding patients' sera were defined based on their general degranulation curve behavior and on the maximal release induced by the allergen (>0.5%). Only sera that responded in the assay at least at a single time point, determined by individual dose-response curves were considered. Nineteen of 47 patients responded in the MRA (n=2 BPE-AC, n=8 BM41, and n=9 placebo). The different treatments were highlighted in the respective colors (BPE-AC=green, BM41=yellow, placebo=red). A cell viability assay was performed per serum (B) and the data were expressed in percentage viability relative to the average of untreated cells (C). For simplicity reasons, only the average of the data derived from the three different time points but from the same patient is shown including standard deviation. The behavior of individual patients in course of the treatment regarding the induction of mediator release upon Bet v 1 cross-linking is shown by connected lines (D). Pearson correlation was performed with MRA data and transformed Bet v 1-specific IgE values (Y=Log[Y]) for the responding patients at the individual time points (t=1, t=2 and t=3, E). AUC, area-under-the-curve; BPE-AC, birch pollen extract-based active comparator. *$p \leq 0.05$.

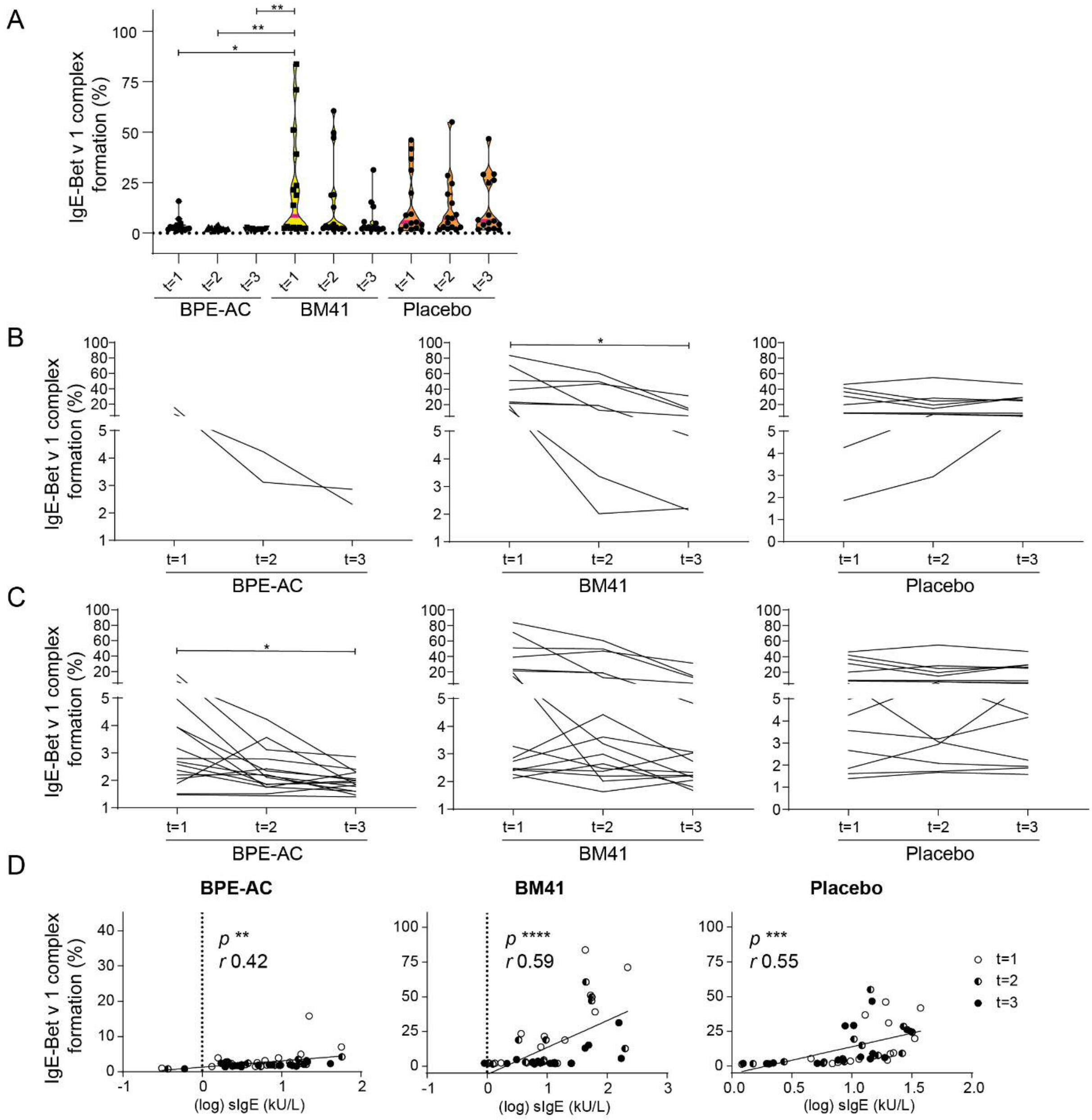
A
IgE-Bet v 1 complex formation (%)
100
75
50
25
0
**
**
*
t=1
t=2
t=3
BPE-AC
BM41
Placebo
B
C
100
80
60
40
20
5
4
3
2
1
0
D
BPE-AC
BM41
Placebo
p **
r 0.42
p ****
r 0.59
p ***
r 0.55
t=1
t=2
t=3
(log) sIgE (kU/L)

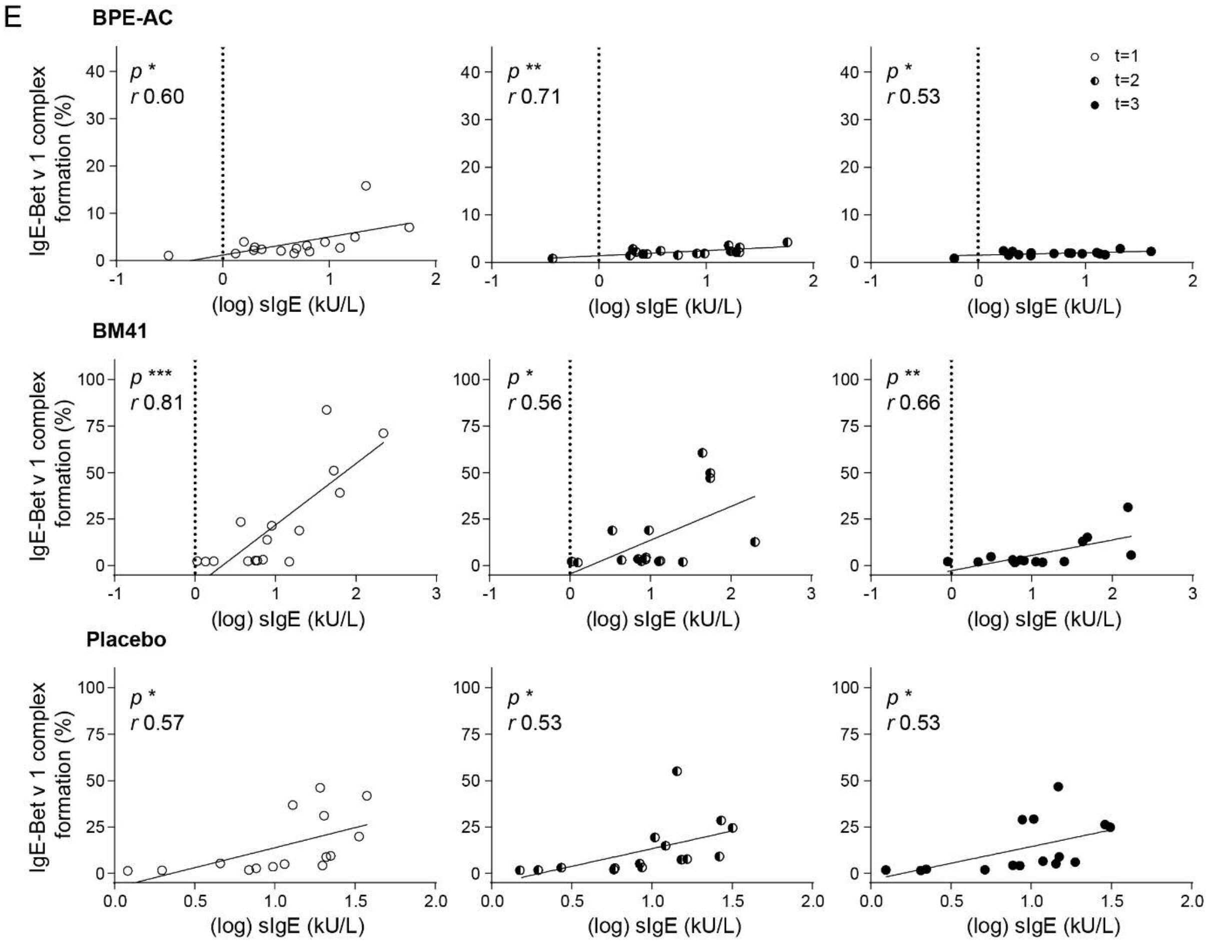

Figure S5 IgE-Bet v 1 complex formation for all patients involved in the study (n=47) before (A, C) and after (B) exclusion of sera based on the responsiveness in the FAB assay as determined by the LOQ. Sera of 22 patients exceeded the LOQ at least at a single time point (n=8 BPE-AC, n=8 BM41, and n=6 placebo). A one-way ANOVA was performed for statistical analysis. Pearson correlation was performed with the FAB data derived from all patients plotted against the transformed Bet v 1-specific IgE levels (Y=Log[Y]) for all time points combined (D) and for the individual time points in separate (E). BPE-AC, birch pollen extract-based active comparator; s, specific. *$p \leq 0.05$; **$p \leq 0.01$; ***$p \leq 0.001$; ****$p \leq 0.0001$.

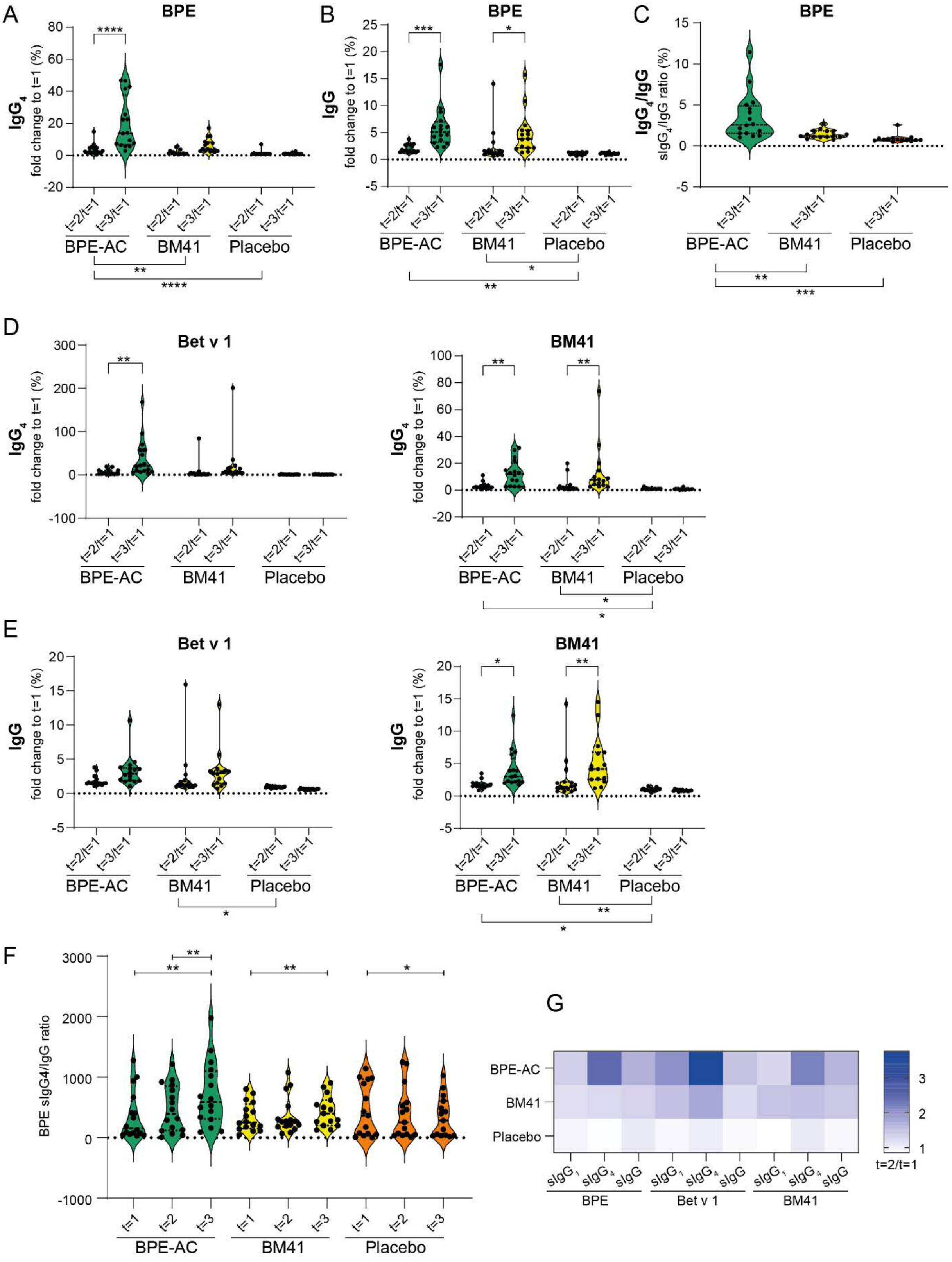

Figure S6 BPE-, Bet v 1- and BM41-specific IgG_4 (A, D) and IgG (B, E) or IgG_4/IgG ratios (C, F) compared to the first time point (t=1). Comparisons of fold increase t=1 to t=2 of BPE-, Bet v 1- and BM41 $sIgG_1$, $sIgG_4$ and sIgG in all three treatment groups shown as median (G). BPE-AC, birch pollen extract-based active comparator. **p* ≤ 0.05; ***p* ≤ 0.01; ****p* ≤ 0.001; *****p* ≤ 0.0001.

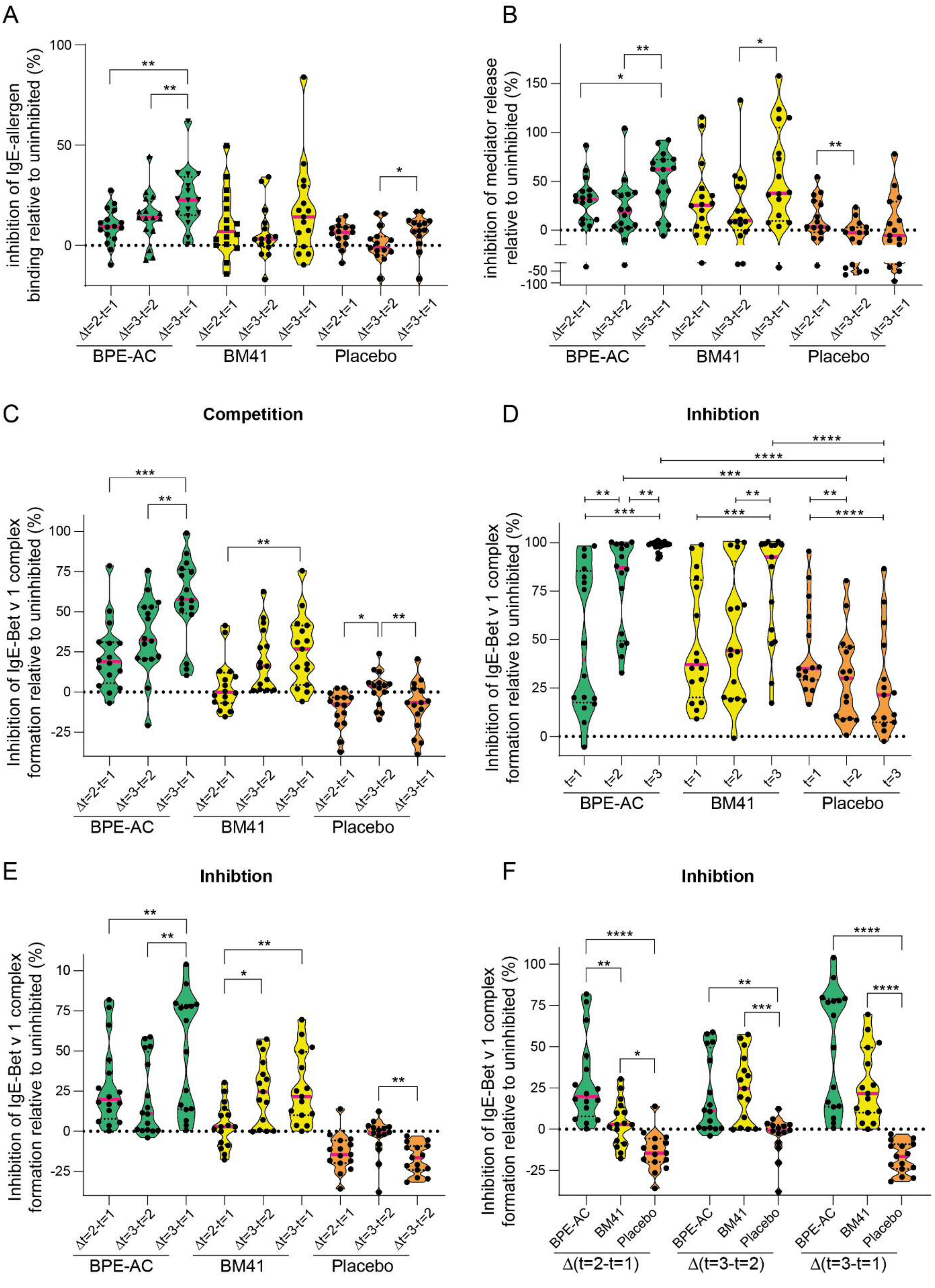

Figure S7 Inter-group comparison of different deltas (t=2-t=1, t=3-t=2, and t=3-t=1) for the inhibition ELISA (A), inhibition of mediator release assay (B) and FABcompetition assay (C) from Figure 3. In addition to the FABcompetition assay, a FABinhibition assay was performed using serum from SCIT-treated subjects (D), and the corresponding delta graphs are shown (E-F). The data were expressed as relative to the uninhibited reference (100%) and are shown as

violin plots with individual data (median is indicated by the red bar). The treatment with BM41 significantly induced the inhibition of IgE-Bet v 1 complex formation over time (t=3-t=1) by Δ55.5% (t=1 37.2%, t=2 44.3% and t=3 92.6%, all medians). In placebo, the inhibition of complex formation even reduced from 35% (t=1) to 21.5% (t=3) by Δ13.6%, likely due to out-of-season decline of Bet v 1-specific IgG. BPE-AC induced with Δ59.2% (t=3-t=1) an even stronger blocking effect than BM41, with a quicker onset (t=1 39.8%, t=2 87.2% and t=3 99%). For placebo, the inhibitory effect even decreased significantly. BPE-AC, birch pollen extract-based active comparator. *$p \leq 0.05$; **$p \leq 0.01$; ***$p \leq 0.001$; ****$p \leq 0.0001$.

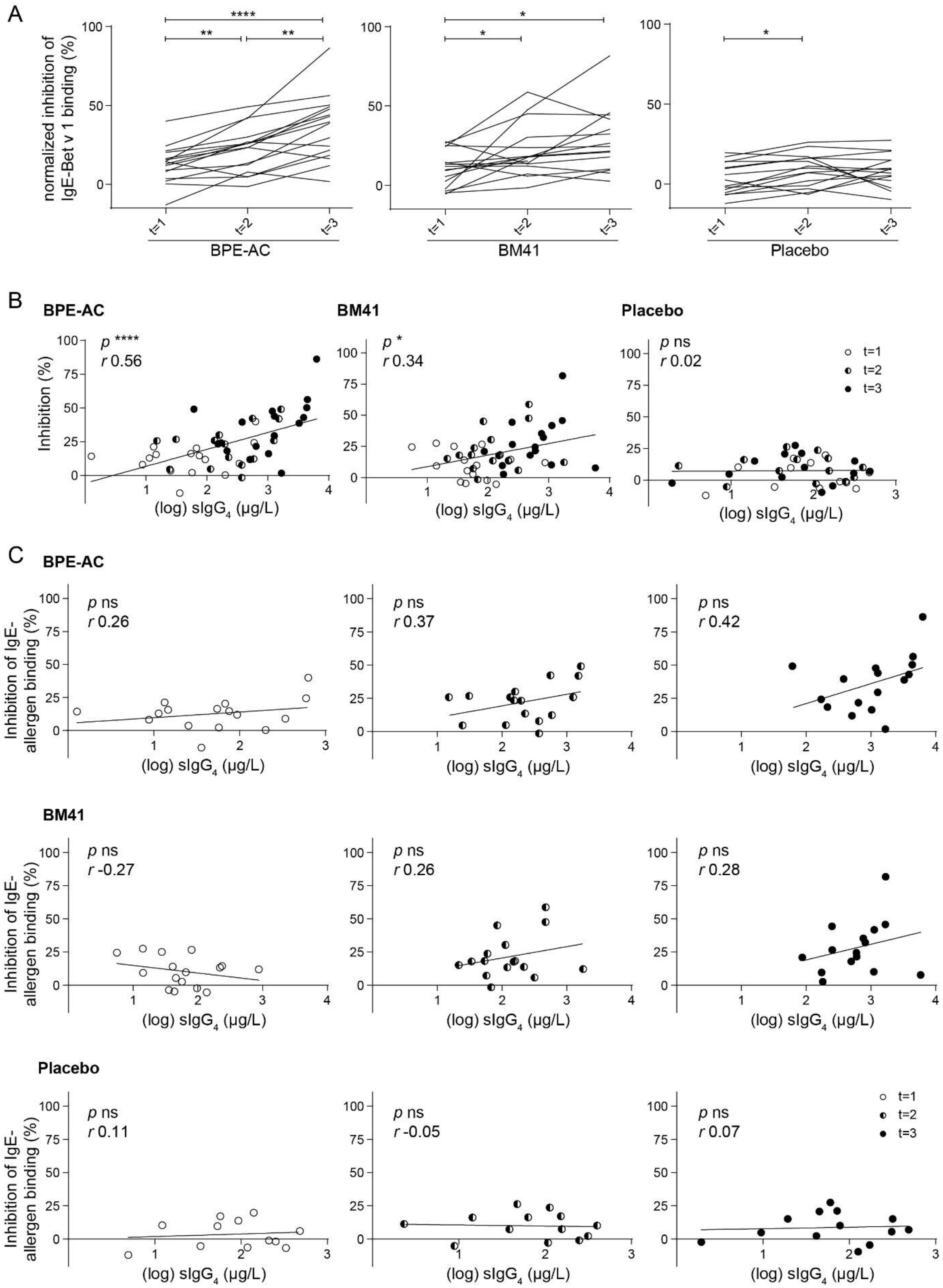

Figure S8 The inhibition of IgE-Bet v 1 binding determined by inhibition ELISA shown for each patient by interconnected lines (A). Pearson correlation for inhibition ELISA data plotted against the transformed Bet v 1-specific IgG_4 levels (Y=Log[Y]) for the three individual time points (t=1, t=2 and t=3, B) or for the individual time points in separate (C). The Bet v 1-specific IgG_4 levels correlated with the inhibition in the active treatment groups (BPE-AC: r = 0.56, and

BM41: $r = 0.34$). This especially became evident when comparing the values of the individual time points with another. Here, Pearson's correlation coefficient increased from 0.26 to 0.42 in the BPE-AC treatment group, and from -0.27 to 0.28 in BM41. In contrast, for placebo the coefficient stagnated around zero. BPE-AC, birch pollen extract-based active comparator; s, specific. *$p \leq 0.05$; **$p \leq 0.01$; ****$p \leq 0.0001$.

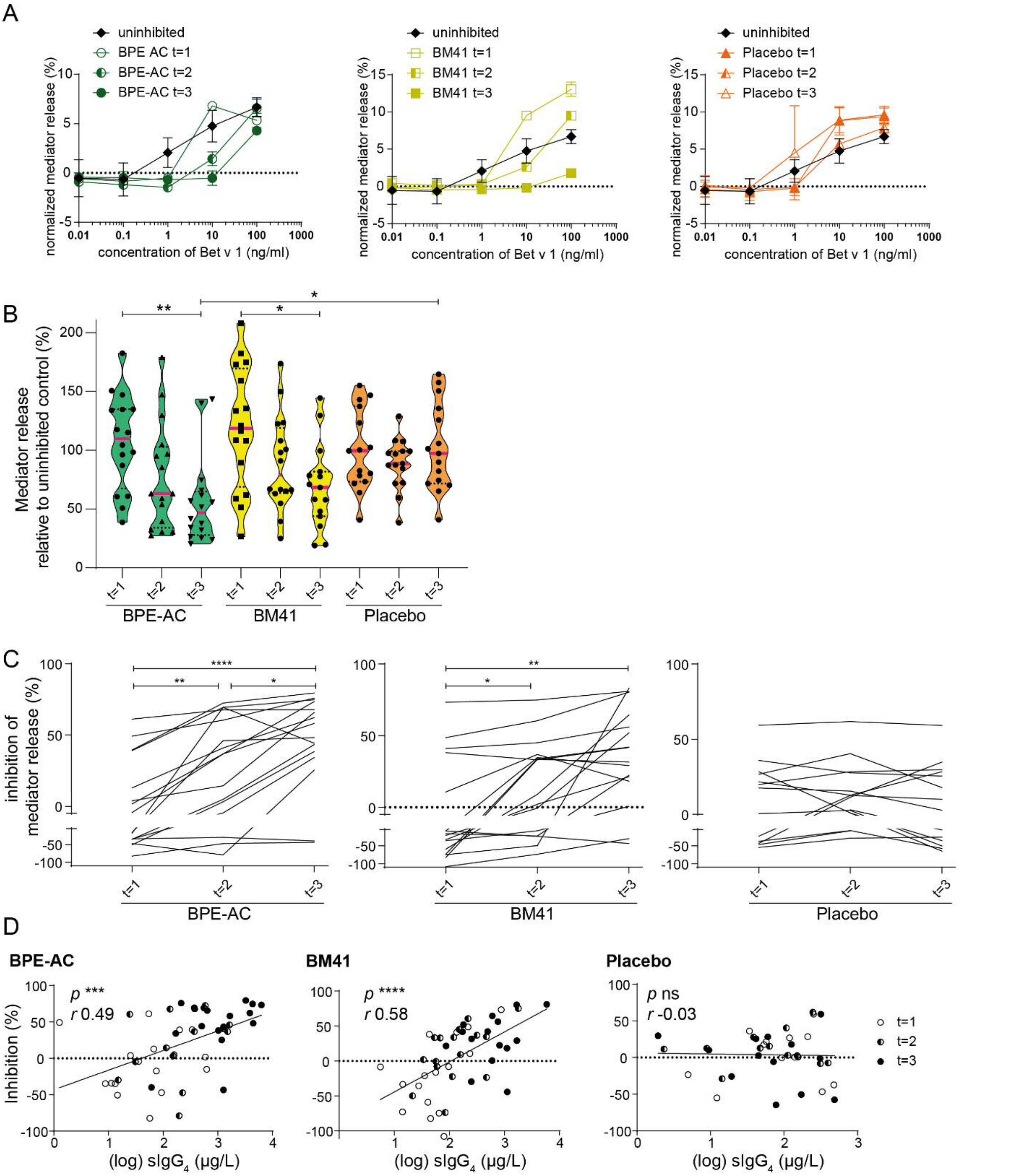

Figure S9 The experimental set-up of the iMRA shown by a representative patient per treatment (A). For the calculation of the total AUC the percentage of degranulation triggered by 1, 10 or 100 ng/ml of Bet v 1 was used. The AUC was expressed as relative to the AUC of the uninhibited control (highlighted in black). AUC of mediator release of each individual patient in course of the treatment prior to calculation of the inhibition (inhibition=100 minus relative iMRA AUC value) shown in Fig 3C-D (B). The behavior of individual patients in course of the treatment regarding the inhibition of Bet v 1-induced mediator release is shown by interconnected lines per treatment (C). Pearson correlation for iMRA data plotted against the transformed Bet v 1-specific IgG_4 levels (Y=Log[Y]) for the three individual time points (t=1, t=2 and t=3) combined (D). Similar to the results of the inhibition ELISA, the inhibitory effect of both active treatments, BM41 and BPE-AC, correlated with Bet v 1-specific IgG_4 levels (r =

0.49, and $r = 0.58$, respectively). No correlation was observed for placebo. BPE-AC, birch pollen extract-based active comparator; s, specific. *$p \leq 0.05$; **$p \leq 0.01$; ***$p \leq 0.001$; ****$p \leq 0.0001$.

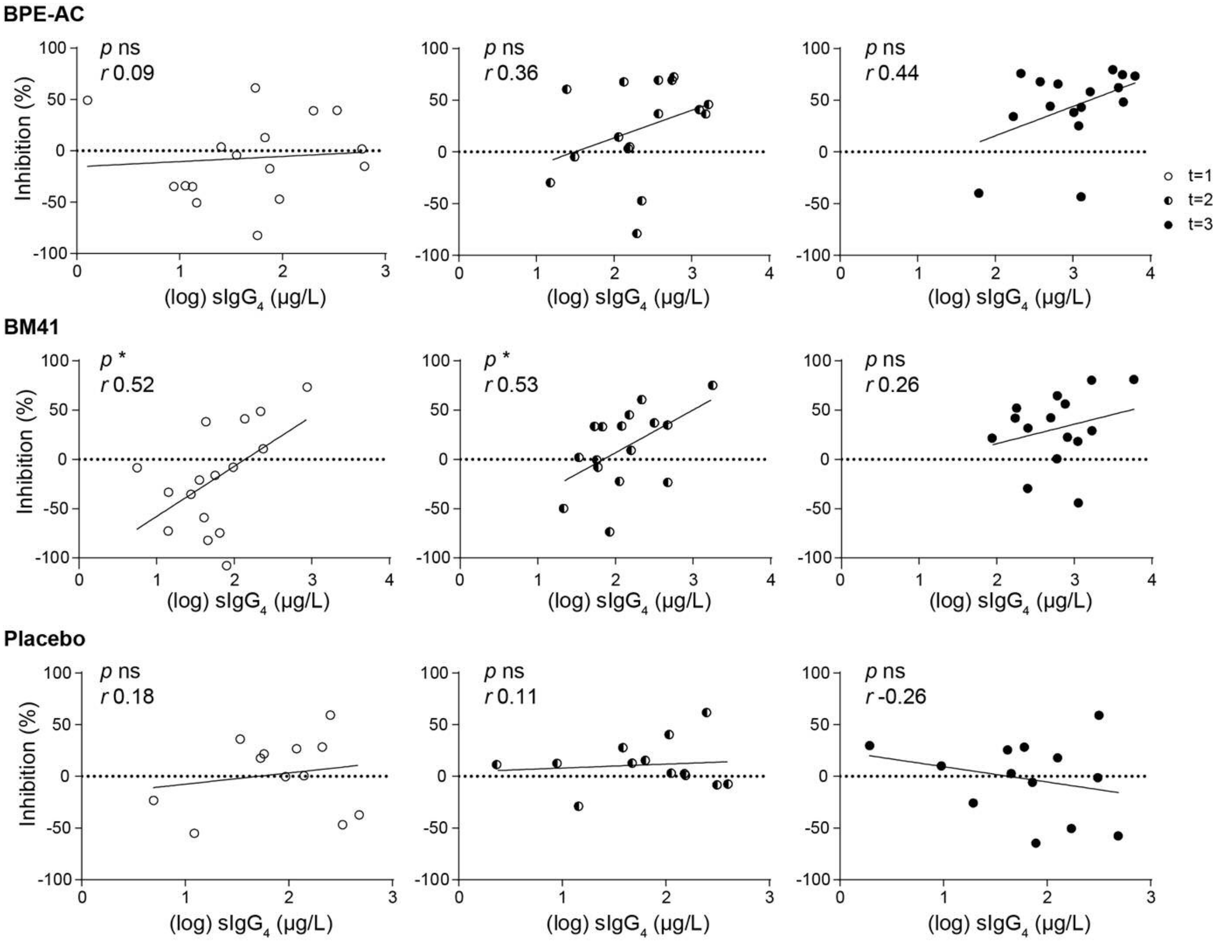

Figure S10 Pearson correlation was performed with iMRA data and transformed Bet v 1-specific IgG_4 values (Y=Log[Y]) at the individual time points of the treatment. Interestingly, in the BPE-AC group rather the later treatment time points correlated with IgG_4 when comparing the individual time points, whereas t=1 did not, implying that functional IgG_4 is induced throughout the treatment. BPE-AC, birch pollen extract-based active comparator; s, specific. $^*p \leq 0.05$.

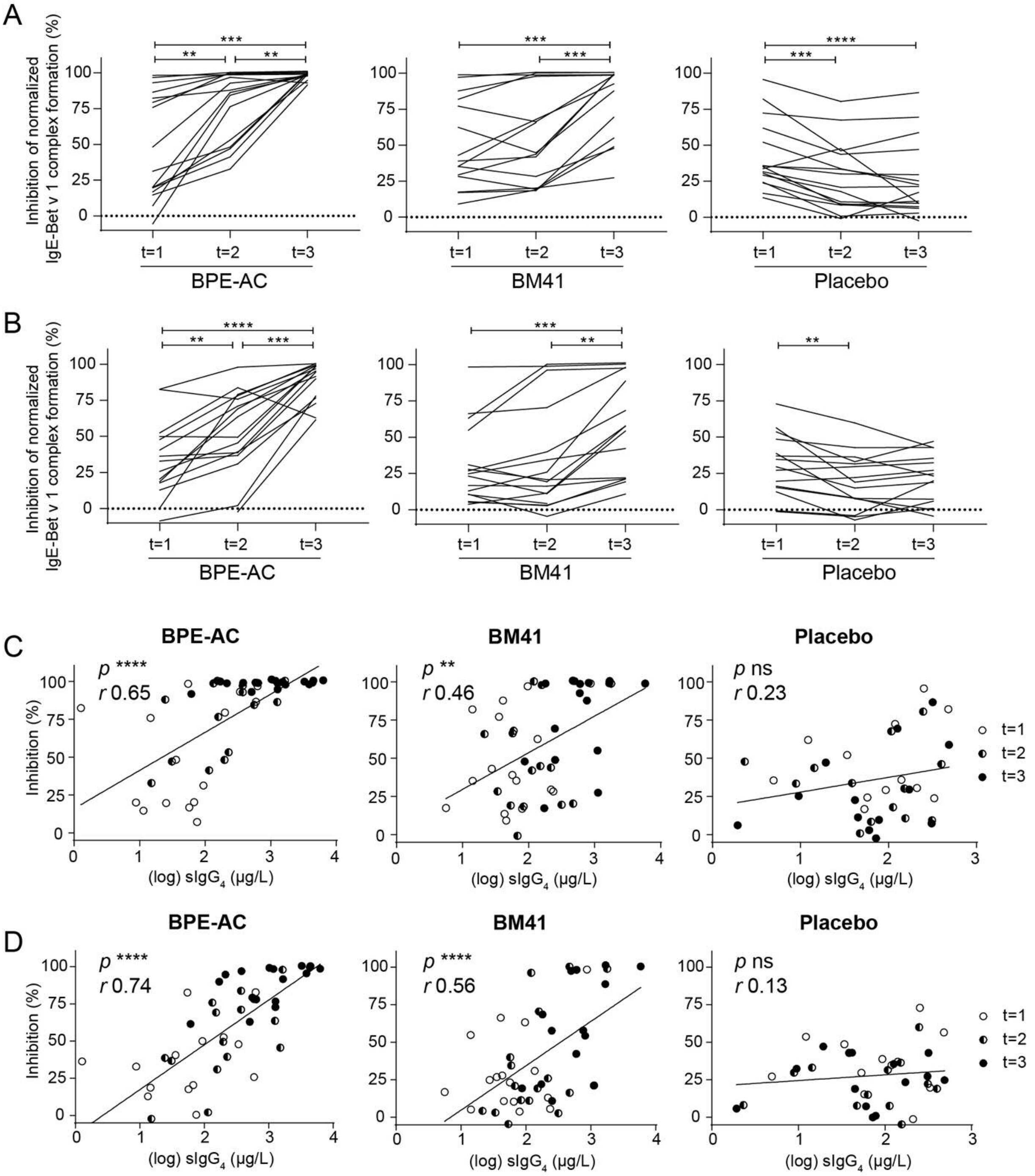

Figure S11 SCIT with BM41 and BPE-AC induces serum inhibitory activity blocking CD23-mediated IgE-facilitated Bet v 1 binding. FABinhibition (A) and FABcompetition (B) assays were performed using serum from SCIT-treated subjects. The data derived from both assays were expressed as relative to the uninhibited reference (100%). The inhibition was calculated by subtracting the individual values per patient from the reference (inhibition=100 minus relative IgE-FAB value). The results for each individual patient is shown in order to follow the treatment-induced changes over time (FABinhibition, A; FABcompetition, B). A Pearson correlation test with transformed Bet v 1-specific IgG_4 data (Y=Log[Y]) showed that the functional blocking is mostly attributed to IgG_4 (C, D, respectively). BPE-AC, birch pollen extract-based active comparator; s, specific. $**p \leq 0.01$; $***p \leq 0.001$; $****p \leq 0.0001$.

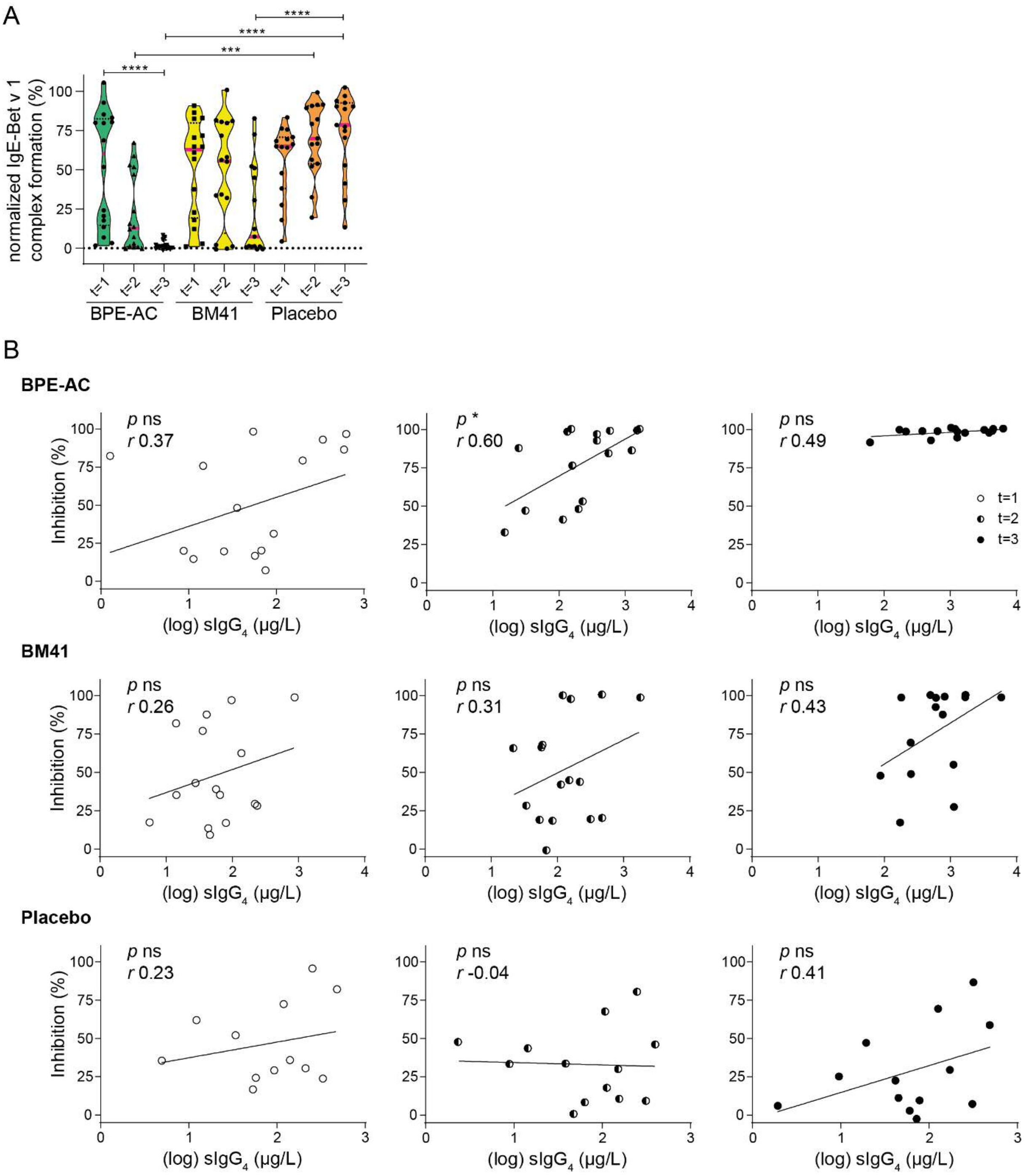

Figure S12 The potential of the patients' sera to intervene with the complex formation of reference IgE and Bet v 1 was monitored over time using the FABinhibition assay and used for the calculation of the inhibition presented in Fig S7D-F (A). Pearson correlation was performed with FABinhibition data and transformed Bet v 1-specific IgG_4 values (Y=Log[Y]) at the individual time points of the treatment (B). BPE-AC, birch pollen extract-based active comparator; s, specific. *$p \leq 0.05$; ***$p \leq 0.001$; ****$p \leq 0.0001$.

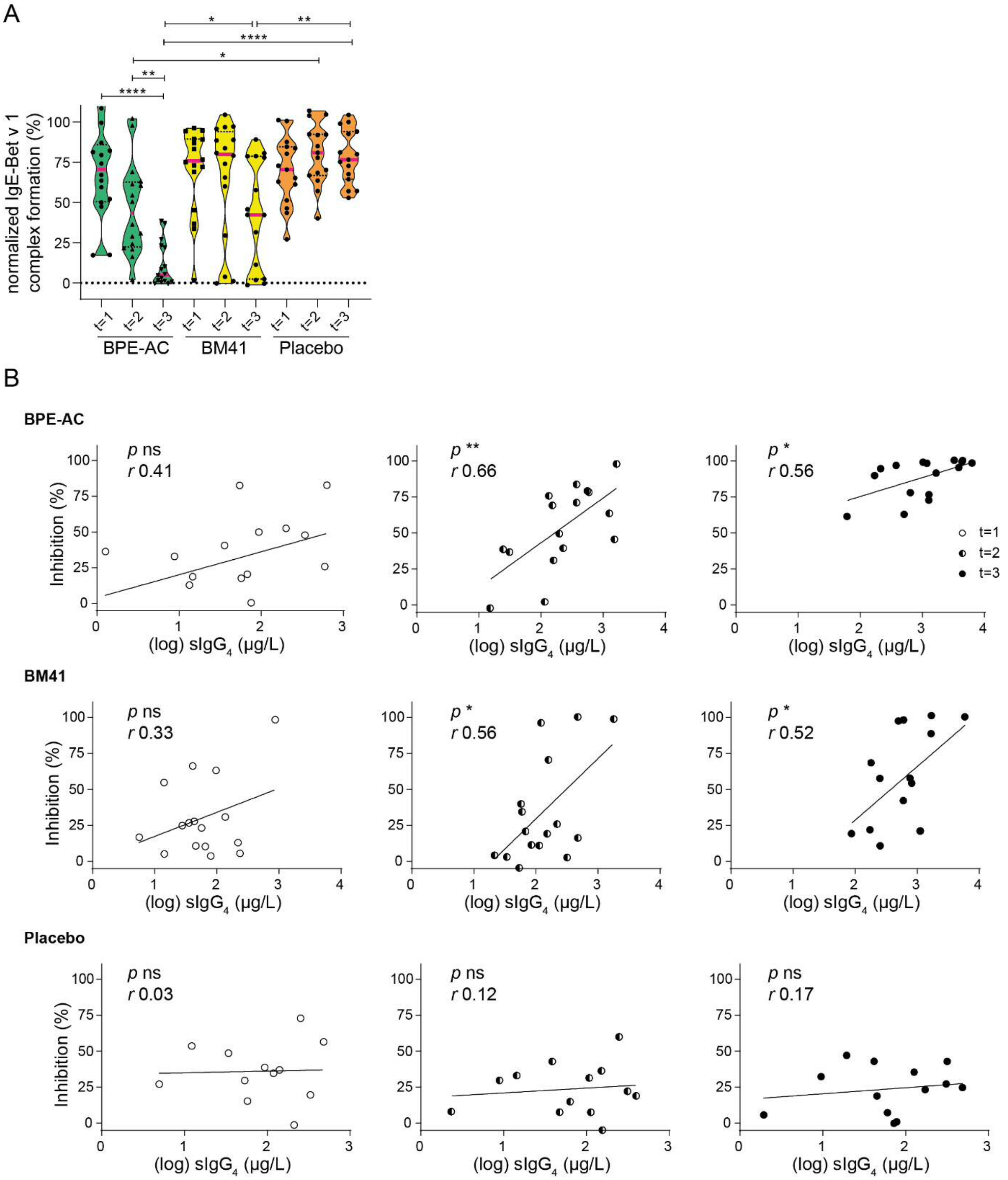

Figure S13 The potential of the patients' sera to intervene with the complex formation of reference IgE and Bet v 1 was monitored over time using the FABcompetition assay and used for the calculation of the inhibition presented in Fig 3E-F (A). Pearson correlation was performed for FABcompetition data plotted against the transformed Bet v 1-specific IgG_4 levels (Y=Log[Y]) for the three individual time points (t=1, t=2 and t=3, B). BPE-AC, birch pollen extract-based active comparator; s, specific. *$p \leq 0.05$; **$p \leq 0.01$; ****$p \leq 0.0001$.

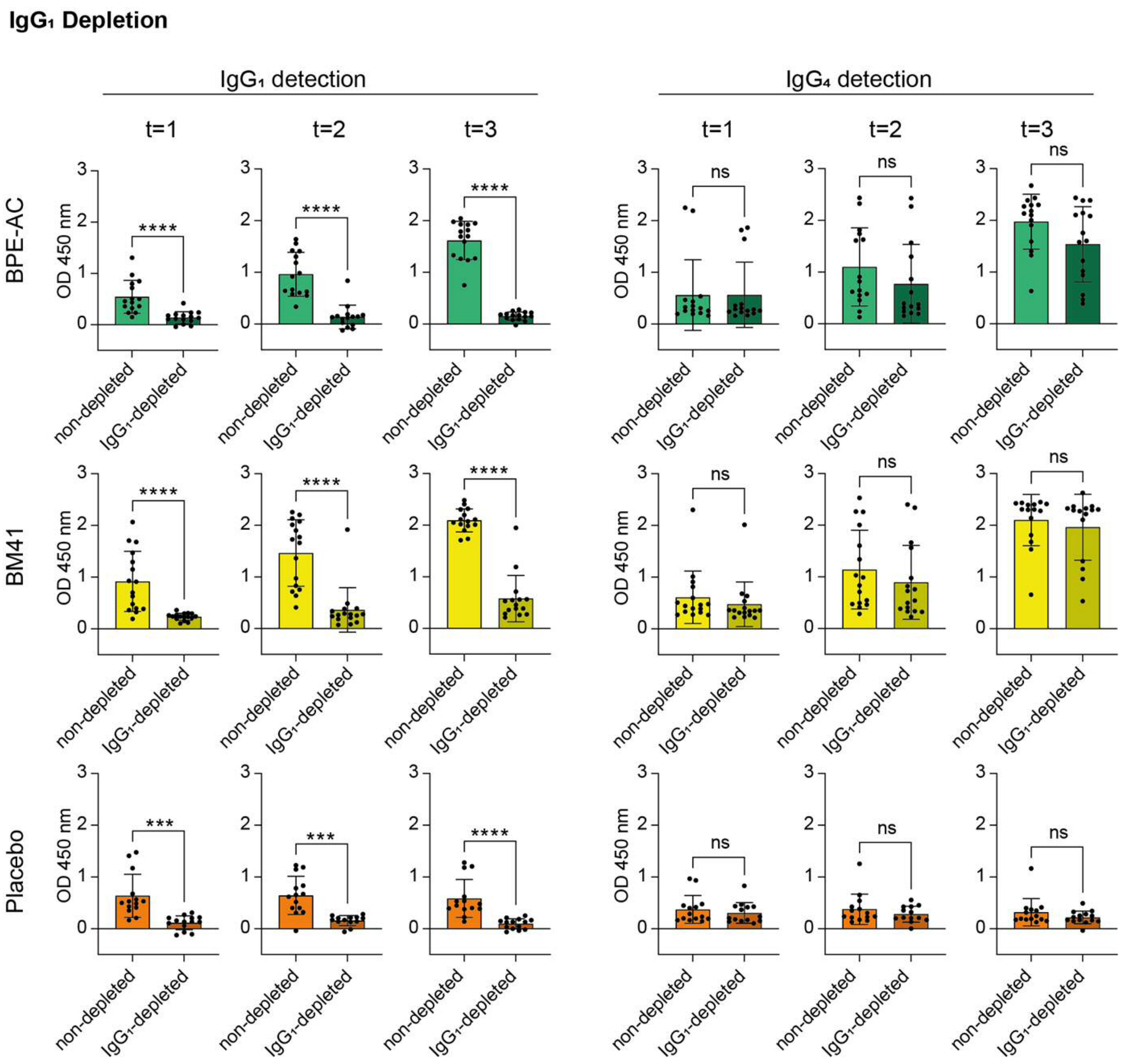

Figure S14 Confirmation that the IgG$_1$ depletion protocol using the CaptureSelect IgG$_1$ human affinity matrix (Thermo Fisher Scientific) is working was done by Bet v 1-specific ELISA. The serum was diluted 1:4 and a mouse anti-human IgG$_1$-Fc HRP antibody (clone HP6001, SouthernBiotech, diluted 1:4000) as well as a mouse anti-human IgG4-Fc HRP (clone HP6025, SouthernBiotech, diluted 1:8000) were used as detection antibodies. Of note, there was not enough serum left of all patients to conduct the depletion protocol, therefore, the depletion was performed with n=15 for BPE-AC, n=16 BM41 and n=14 placebo. This also refers to Fig.S15, S16 and S17, as well as Fig.5 of the main text. BPE-AC, birch pollen extract-based active comparator. ***$p \leq 0.001$; ****$p \leq 0.0001$.

IgG_4 Depletion

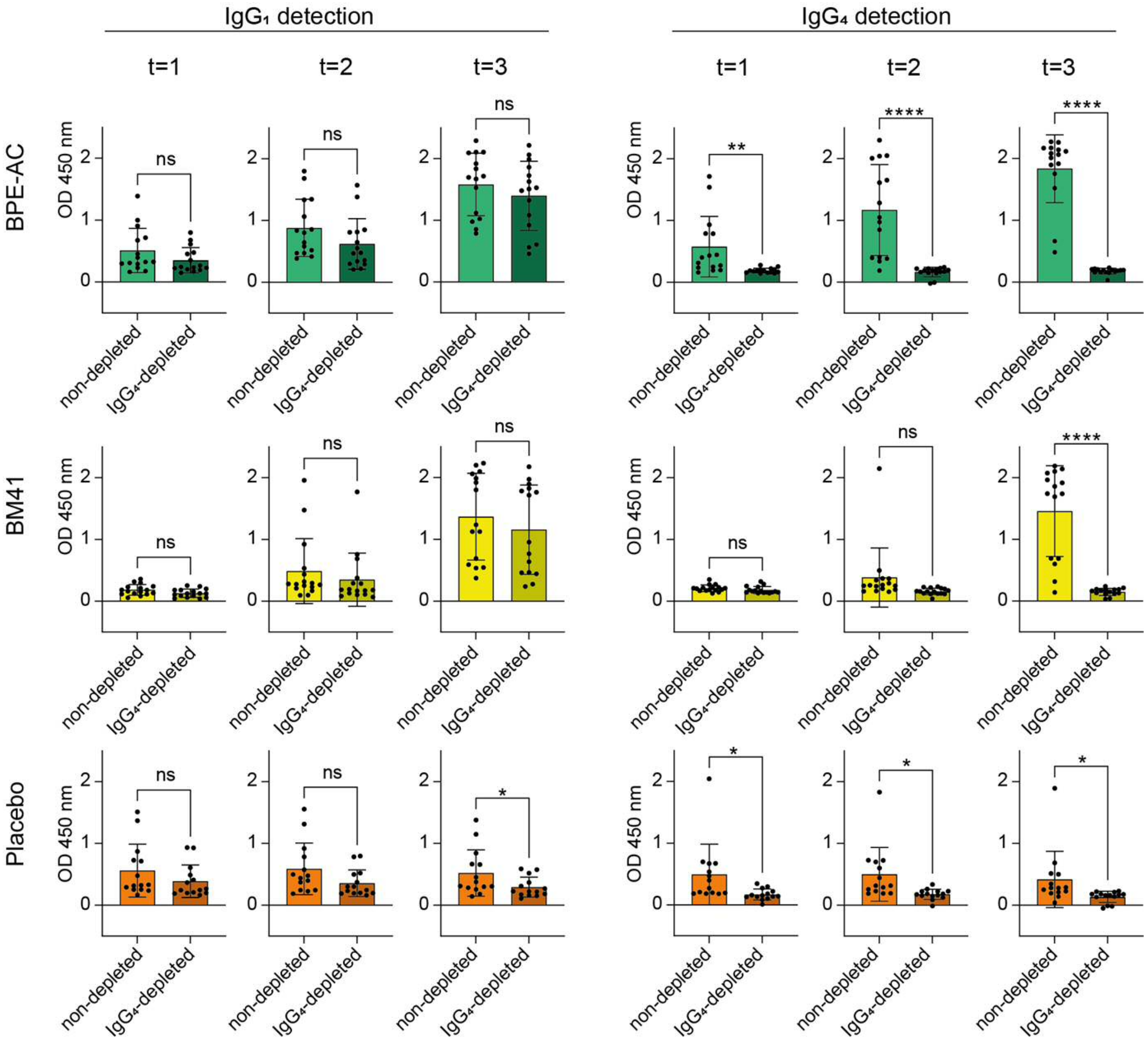

Figure S15 Validation of the IgG_4 depletion protocol by Bet v 1-specific ELISA. For the depletion of IgG_4 the CaptureSelect IgG_4 human affinity matrix (Thermo Fisher Scientific) was used. For the ELISA, the same serum dilution of 1:4 and the same antibodies as stated in the legend of Figure S14 were used. BPE-AC, birch pollen extract-based active comparator. *$p \leq 0.05$; **$p \leq 0.01$; ****$p \leq 0.0001$.

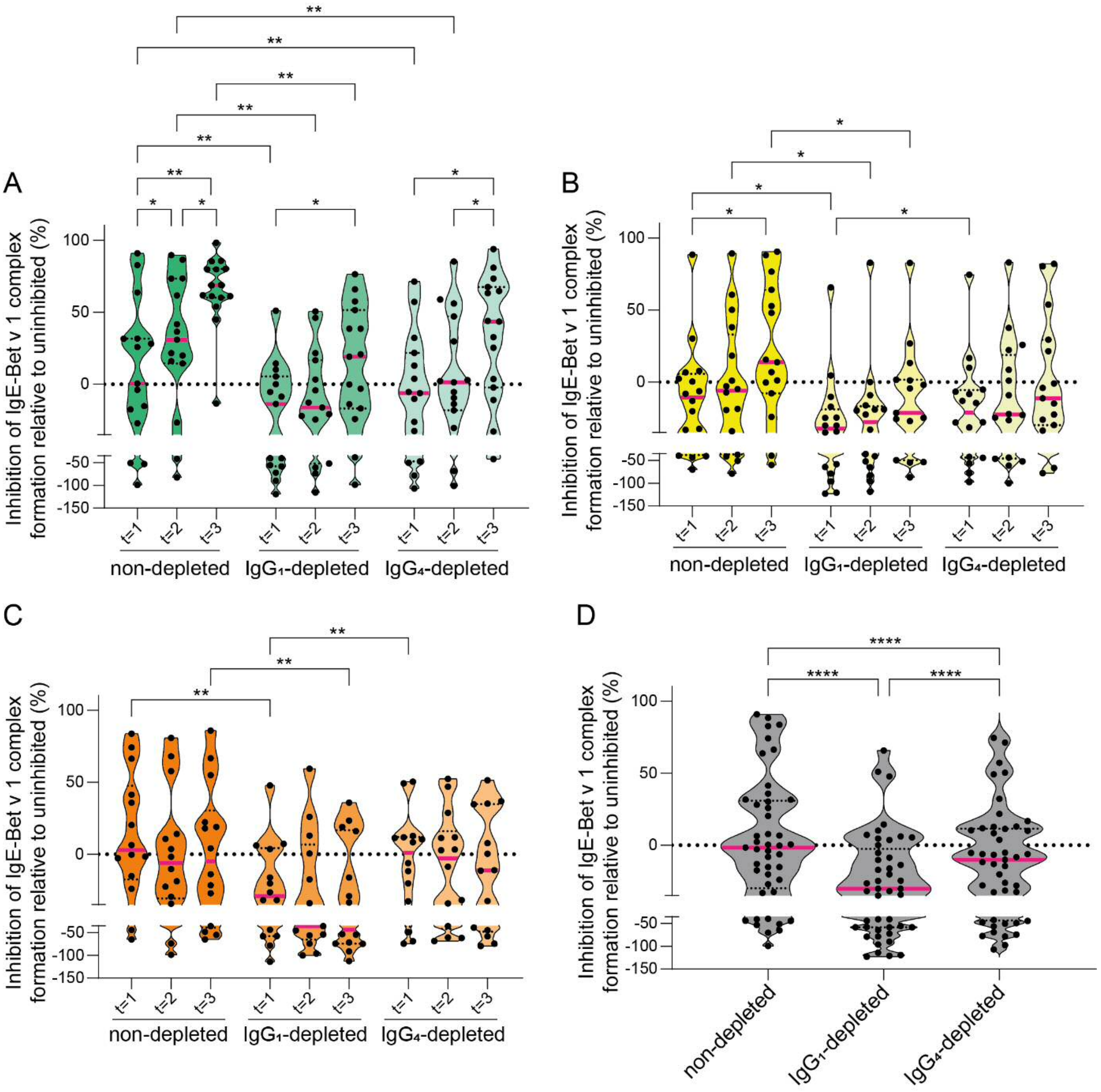

Figure S16 Data of FABinhibition assay of the BPE-AC (A), BM41 (B) and placebo group (C) performed using the non-, IgG_1- and IgG_4-depleted sera that were used to calculate the delta values shown in Figure 5A. T=1 data points of all groups combined show that IgG_1 is mainly responsible for serum inhibitory activity before the treatment and not IgG_4 (D). $^{*}p \leq 0.05$; $^{**}p \leq 0.01$; $^{****}p \leq 0.0001$.

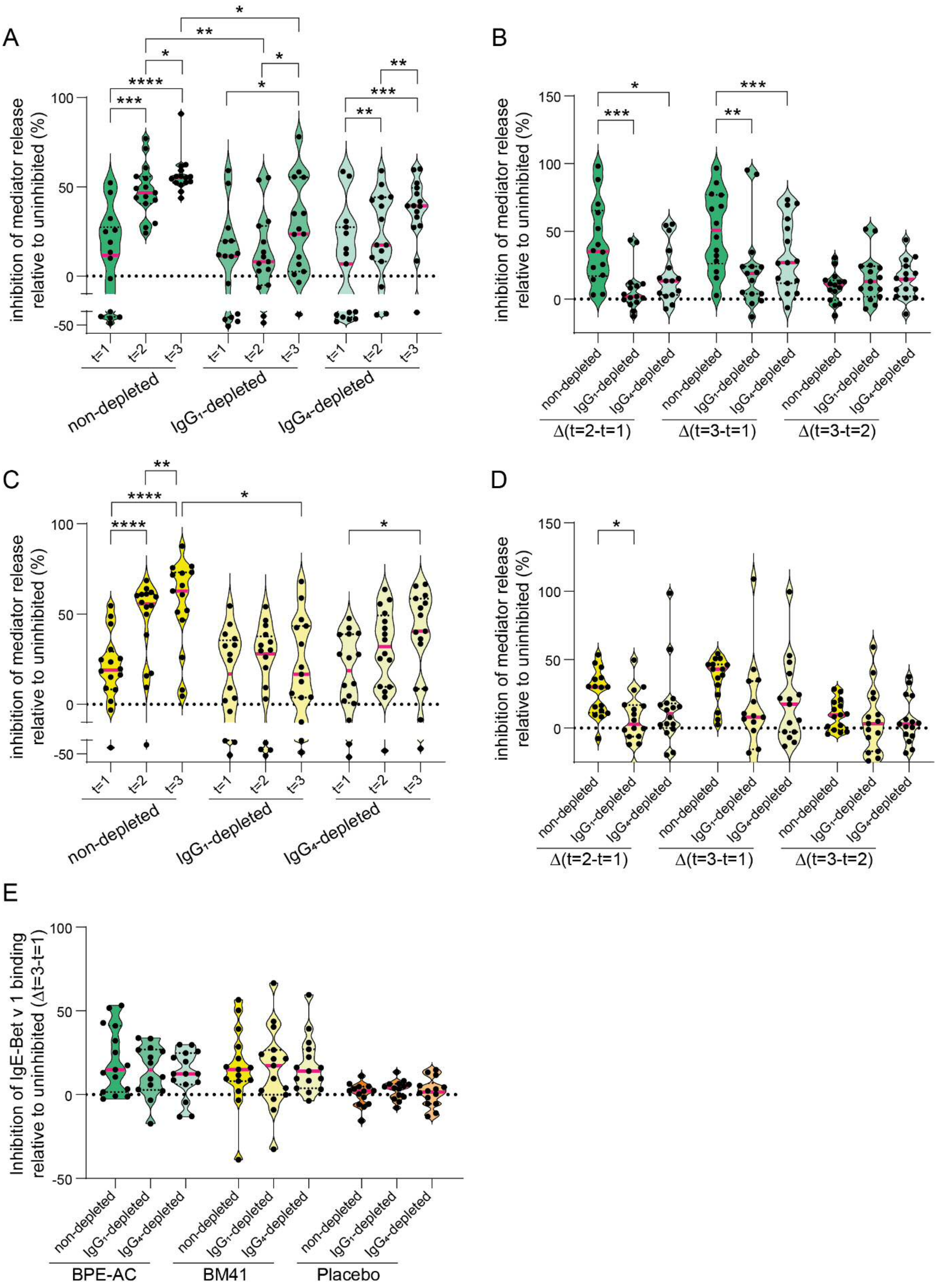

Figure S17 Inhibition of mediator release of the BPE-AC (A) and BM41 (C) group performed using the non-, IgG_1- and IgG_4-depleted sera that were used to calculate the delta values shown in Figure 5B, and corresponding delta graphs (B and D). Of note, of one non-depleted serum of the BPE-AC group not enough serum was left to conduct the mediator release assay, therefore, here the sample number was n=14. This also refers to Fig.5B and D of the main text.

Competition ELISA using a set of two Bet v 1-specific IgE antibodies that were simultaneously incubated with the IgE-depleted patients' sera (either IgG-non-depleted, IgG_1-depleted or IgG_4-depleted) in a 1:4 dilution (E). The data were expressed as relative to the uninhibited reference (100%) and are shown as delta t=3-t=1. BPE-AC, birch pollen extract-based active comparator. $*p \leq 0.05$; $**p \leq 0.01$; $***p \leq 0.001$; $****p \leq 0.0001$.

References

1. Aglas L, Bethanis A, Chrusciel P, Stolz F, Gruen M, Jaakkola UM, et al. In vivo Induction of Functional Inhibitory IgG Antibodies by a Hypoallergenic Bet v 1 Variant. Front Immunol 2020; 11:2118.
2. Wallner M, Hauser M, Himly M, Zaborsky N, Mutschlechner S, Harrer A, et al. Reshaping the Bet v 1 fold modulates T(H) polarization. J Allergy Clin Immunol 2011; 127:1571-8.e9.
3. Pichler U, Asam C, Weiss R, Isakovic A, Hauser M, Briza P, et al. The fold variant BM4 is beneficial in a therapeutic Bet v 1 mouse model. Biomed Res Int 2013; 2013:832404.
4. Soh WT, Aglas L, Mueller GA, Gilles S, Weiss R, Scheiblhofer S, et al. Multiple roles of Bet v 1 ligands in allergen stabilization and modulation of endosomal protease activity. Allergy 2019; 74:2382-93.
5. Shamji MH, Ljørring C, Francis JN, Calderon MA, Larché M, Kimber I, et al. Functional rather than immunoreactive levels of IgG4 correlate closely with clinical response to grass pollen immunotherapy. Allergy 2012; 67:217-26.
6. Pena-Castellanos G, Smith BRE, Pomés A, Smith SA, Stigler MA, Widauer HL, et al. Biological activity of human IgE monoclonal antibodies targeting Der p 2, Fel d 1, Ara h 2 in basophil mediator release assays. Front Immunol 2023; 14:1155613.
7. Strobl MR, Demir H, Sánchez Acosta G, Drescher A, Kitzmüller C, Möbs C, et al. The role of IgG(1) and IgG(4) as dominant IgE-blocking antibodies shifts during allergen immunotherapy. J Allergy Clin Immunol 2023; 151:1371-8.e5.
